# Supplementary material for: Effectiveness of erectogenic condom against semen exposure among women in Vietnam: Randomized controlled trial
Source: PLoS One. 2022 Feb 17;17(2):e0263503. doi: 10.1371/journal.pone.0263503 (PMC8853499; doi:10.1371/journal.pone.0263503)
Supplement: S1 File — (PDF) [file pone.0263503.s002.pdf]

Promoting CSD500 Use among Women in Established Relationships

Protocol

Jan 15, 2017

Collaborating institutions:

The Ohio State University, Columbus, OH, U.S.
Ministry of Health of Vietnam
University of North Carolina at Chapel Hill, NC, U.S.

Short study title: "Vietnam CSD500 Study" or "VN CSD500"

TABLE OF CONTENTS

ABBREVIATIONS AND ACRONYMS 3
1. ADMINISTRATIVE INFORMATION 4
2. STUDY SUMMARY 5
3. STUDY PRODUCT: CSD500 5
4. RATIONALE 6
5. PSA OUTCOME 8
6. STUDY POPULATION 9
7. STUDY AIMS 10
8. STUDY PROCEDURES 11
9. STATISTICAL CONSIDERATIONS 13
10. DATA MANAGEMENT 16
11. TRANSLATION 16
12. INCIDENTS AND INCIDENT REPORTING 16
13. PROTECTION OF HUMAN SUBJECTS 18
14. PLANS FOR REPORTING RESULTS 19
15. REFERENCES 20
16. ATTACHMENTS 24

ABBREVIATIONS AND ACRONYMS

AE	Adverse event
CCTS	Center for Clinical and Translational Science
CI	Confidence interval
DCF	Data collection form
DSMC	Data Safety Monitoring Committee
GEE	Generalized estimating equation
IRB	Institutional review board
MOH	Ministry of Health
OSU	The Ohio State University
PBS	Phosphate buffered saline
PI	Principal investigator
PIN	Participant identification number
PSA	Prostate-specific antigen
QSE	Quality of Sexual Experience
RCT	Randomized controlled trial
RR	Relative risk
SAE	Serious adverse event
SEX-Q	Sexual Experience Questionnaire
SRPS	Sexual Relationship Power Scale
STD-CRC	Sexually Transmitted Diseases Cooperative Research Center
UNC	University of North Carolina
UPIRTSO	Unanticipated problem involving risk to subjects or others
USD	United States dollar
VND	Vietnamese dong

1. ADMINISTRATIVE INFORMATION

1.1 Key staff

Study investigators and primary affiliations are as follows:

Investigator	Institution	Study role
John Casterline, PhD	OSU	Co-investigator
Marcia Hobbs, PhD	UNC	Co-investigator
Maria Gallo, PhD	OSU	Principal Investigator
Nghia Nguyen, MD, PhD	MOH-Vietnam	Site Investigator
Rebecca Andridge, PhD	OSU	Statistician

1.2 Study organization

The study will be fielded at the Thanh Hoa Provincial Centre of Reproductive Health in Vietnam. Testing for prostate-specific antigen (PSA) will be conducted at the Microbiology and Human Challenge Core Laboratories of the UNC Sexually Transmitted Diseases Cooperative Research Center (STD-CRC) in Chapel Hill, NC, US. Data management will occur through collaboration between the field site and OSU. The Site Investigator will manage the daily trial operation. Key staff will employ regular communication by telephone (occurring weekly during study start-up and initiation and then decreasing to a monthly basis unless needed more often), and the Principal Investigator (PI) or designee will perform on-site monitoring at pre-initiation, during study implementation (occurring annually or more often, if needed), and at study close-out.

1.3 Ethical review

Ethical oversight for the research will be provided by The OSU Biomedical Institutional Review Board (IRB), which is registered with the Office of Human Research Protections and covered by a Federal Wide Assurance (FWA00006378), and the Hanoi University of Public Health Biomedical IRB (FWA00009326). UNC-Chapel Hill will defer their review of the protocol to the OSU IRB.

2. STUDY SUMMARY

This randomized controlled trial will test whether promoting the novel condom CSD500 (Futura Medical Developments; Surrey, UK) for improved sexual pleasure results in couples having less unprotected vaginal sex – measured with a biological marker of recent semen exposure – compared to the promotion of a standard condom for disease and pregnancy prevention. CSD500 contains an erectogenic drug and was developed to improve sexual pleasure by increasing penile firmness, size, and erection duration.

We will recruit eligible, monogamous, heterosexual couples at Thanh Hoa Provincial Centre of Reproductive Health in Vietnam to participate in a randomized controlled trial (RCT) of two balanced arms of approximately 250 couples each. At enrollment and at three follow-up visits scheduled at 2, 4 and 6 months after enrollment, we will provide condom counseling, will collect double-headed vaginal swabs to test for the semen biomarker, prostate-specific antigen (PSA), and will administer a questionnaire to the female participants on their demographics, sexual and condom-related attitudes and practices, including sexual pleasure and perceptions of partner's sexual pleasure. Although the intervention is directed to the women, couples will be enrolled to ensure that the male partners consent to CSD500 use. After enrollment, male participants will not participate in study visit activities, including data collection, until the time of their female partner's final 6-month visit. Because of the differences in counseling messages, participants and study staff at the site cannot be blinded to arm assignment.

Primary aims:	1. To evaluate whether CSD500 provided to improve sexual pleasure, as compared to the standard condom, results in less semen exposure among women in an established relationship<br>2. To evaluate the effects of CSD500 provision on men and women's reports of sexual pleasure compared to the standard condom<br>3. To identify correlates of semen exposure measured by PSA positivity
Study design:	RCT with two arms: CSD500 for sexual pleasure or the standard condom currently provided for pregnancy and disease prevention
Study site:	Thanh Hoa Provincial Centre of Reproductive Health
Data collection:	Women: questionnaires and vaginal swabs for PSA testing at enrollment and the 2, 4, and 6-month visits<br>Men: questionnaires at enrollment and the 6-month visit
Sample size:	~500 couples with balanced assignment to the two study arms
Study population:	Adult, monogamous, heterosexual couples willing to use the assigned condoms and who at enrollment are not using – or intending to start in the next 6 months – a modern contraceptive method other than condoms
Data analysis:	Intent-to-treat analysis

3. STUDY PRODUCT: CSD500

CSD500 is a commercial condom that complies fully with the requirements of ISO 4074, the international standard for male latex condoms. The 53 mm-width, latex condom is transparent with a teat at the end containing a pharmacological dose of Zanifil™ (active ingredient glyceryl trinitrate 1% w/w). The product works by increasing blood flow, which helps to maximize erection hardness.

CSD500 has been approved for over-the-counter use in 27 European nations and is being sold under the name "*Blue Diamond*™" in The Netherlands and Belgium but has not been registered in the U.S. or Vietnam. They will be labeled with the brand name "Futura Max" in the present trial. We will meet NIH requirements for regulatory oversight (see http://www.niaid.nih.gov/researchfunding/sci/human/pages/hshandbook.aspx) by obtaining regulatory approval from the Vietnam MOH to use the product solely for research purposes in compliance with local laws (see letter from Dr. Hong). Futura Medical Developments has agreed to provide CSD500 for the study at no cost without having any other role or influence (see letter from David Davies). To maximize sales and improve adherence to condom use, the manufacturer recommends that its distribution partners use a target price that is, at most, 50% more than current condom prices (David Davies, written communication, August 2014). CSD500 is expected to be widely available in settings worldwide, and its access is unlikely to be limited by price given its modest premium compared to standard male condoms.

Two industry-sponsored trials have assessed the effects of CSD500 on user performance and pleasure. The first was a randomized crossover trial conducted among 120 healthy, heterosexual couples recruited from a clinic in London, England who reported being in stable relationships of ≥6 months and in which the male partner reported ≥1 partial or total loss of penile tumescence during condom use in the past six months (David Davies, written communication, August 2014). Couples were randomly assigned to use CSD500 or standard condoms for five acts followed by five uses of the other condom type. Both the man and woman independently completed a diary for each condom use. Females perceived an increase in penile firmness ($p<0.01$), an increase in penis size ($p=0.07$), and erections of longer duration ($p=0.04$) with CSD500 relative to the standard condom. Men also reported increased penis size ($p<0.01$) and erections of longer duration ($p=0.02$) with CSD500 compared to the standard condom. The second was a single-arm trial of CSD500 among heterosexual men (N=91) and women (N=38) in Argentina. Both men (54%) and women (63%) reported that the condom improved sexual performance, with participants citing improved sensations, comfort, texture, erection duration and confidence (James Barder, written communication, January 2015).

4. RATIONALE

Promoting a novel condom designed to improve sexual pleasure could lead to less unprotected sex and, thereby, reduce the risks of both unintended pregnancy and HIV/STI.

As the sole method for dual protection against pregnancy and HIV/STI, condoms play an important role in public health. Male condoms protect against pregnancy (Trussell 2011) and the transmission of HIV, chlamydia, gonorrhea, trichomoniasis, herpes simplex virus type 2, and syphilis (Holmes 2004; Weller 2002). Given their effectiveness against both pregnancy and disease when used consistently and correctly, condoms alone (rather than with another modern method) have been recommended for those needing dual protection (O'Leary 2011). Condoms have many positive attributes: low cost, availability without a prescription, minimal side effects, few contraindications, and global presence. Sustained use, however, is notoriously difficult to achieve. Only 9% of reproductive-age women in the U.S. in 2011-2013 reported current condom

use (Daniels 2014). Similarly, a national survey in Vietnam found that only 9% and 7% of sexually-active, married, reproductive-age women reported condom use at last sex act and consistent condom use, respectively (Do 2011). Substantial unmet need for contraception, partly stemming from concerns about safety of hormonal contraception, drives Vietnam's high abortion rate. Married women account for about one-third of HIV cases in Vietnam (MOH 2009), and among this group, most were exposed to the virus through sexual activity with their husband (UNAIDS 2010; Nguyen 2008). **Thus, increasing condom use within established partnerships is crucial for preventing HIV/STIs while also protecting women against unintended pregnancy.**

A range of condom-related factors can diminish sexual pleasure: physical effects, including dulled sensation, pain, discomfort, or vaginal dryness (possibly from inadequate condom lubrication); unappealing perception of the touch, taste, or smell of condoms; lack of spontaneity or interruption to the flow of sex; and social constructs (e.g., decreased emotional closeness or inability to express male vitality by ejaculating into the vagina) (Hensel 2012; Sanders 2014). **Dulled sensation can lead to erection loss: an estimated 9%-37% of condoms users have had erection problems during condom application or use** (Graham 2006; Sanders 2012; Sanders 2014). This experience – especially during initial, formative sexual encounters – has been shown to make some men less confident about their ability to maintain an erection during condom use, which may then cause a negative feedback loop of erectile dysfunction (Sanders 2014). Condom-related erection loss also could dissuade men from future attempts to use a condom.

A national survey in Vietnam found that despite widespread lack of personal experience with the device, about 70% of youth believed that condoms reduce sexual pleasure (MOH 2005). This finding is consistent with a 14-country study identifying diminished sexual pleasure as the primary objection to condom use (Mehryar 1995) and with a large U.S. study, in which 31% of heterosexual male and female users of condoms reported a problem with the feel of the device (Crosby 2013). Although the physical properties of condoms directly affect men, women can be even more resistant toward condom use possibly, in part, from weighing their male partner's pleasure as an important contributor toward their own pleasure (Higgins 2008). Women also might be reluctant to require condom use because of concerns that this will lead to their partner seeking unprotected sex elsewhere. Perceptions about condom effects on sexual pleasure and arousal are important because they are directly related to frequency of actual use (Crosby 2013; Higgins 2009; Randolph 2007). While the effectiveness of structural and community-level interventions for increasing condom use remains unclear (Moreno 2014), individual counseling has been shown to reduce STI incidence among adolescent and high-risk adult populations (Lin 2008). Less research has focused on heterosexual couples in purportedly monogamous relationships.

Increasingly, the importance of sexual pleasure has been recognized for informing interventions on safer sex. Condoms have been modified in terms of their size, taste, appearance, texture, smell and thickness to improve the user experience (Cecil 2010; Crosby 2010). Eroticizing the protection afforded by condoms appears to be a successful strategy employed by consistent condom users in Mexico and the Dominican Republic (García 2003; García 2004). Other interventions have included selling condoms packaged with water-based lubricant sachets as pleasure enhancements in Cambodia and socially-marketing textured male condoms in Uganda (Philpott 2006). Increased sales of these products suggest that interventions to improve sexual pleasure with condoms can increase frequency of condom use.

5. PSA OUTCOME

The primary study endpoint, detection of PSA in vaginal fluid, is a feasible marker of women's recent exposure to semen from penile-vaginal sex. Forensic research demonstrates that PSA clears from the vagina in a mean of 20-27 hours and is undetectable after 47 hours (Graves1985; Kamenev 1989). More recently, the protein has been studied for its potential as a semen biomarker for research purposes. Three studies using a controlled design in which women were exposed to specific quantities of their partner's semen in a clinic setting showed high proportions of women tested PSA-positive immediately after exposure (Lawson 1998) followed by a sharp decay curve (Macaluso 1999; Jamshidi 2013). For example, when women were exposed to 1 mL of semen (a volume on the same order of magnitude as the amount in a typical ejaculate) (Cooper 2010), Macaluso et al. found PSA (>1 ng/mL) in 92%, 29% and 3% of women immediately, 24 hours, and 48 hours afterwards, respectively (Macaluso 1999). Given this pattern of clearance from vaginal fluid, observed PSA positivity should be interpreted as the lower bounds of exposure in the past 48 hours. PSA is stable in specimens stored at −70°C after collection (Gallo 2013a). Because it is expressed independently of spermatozoa, PSA is useful for identifying exposure even from men who are vasectomized or otherwise without high levels of spermatozoa. False positive PSA tests from women's endogenous sources of PSA (e.g., serum or urine) are improbable given that the resulting PSA levels would be several orders of magnitude below the established threshold for defining PSA positivity from semen exposure (Gallo 2013b).

To compare condom use between randomization arms, we will sample three short periods of exposure during follow-up (at 2, 4 and 6 months). Studies relying on self-reports of sex and condom use have a similar limitation in that they rarely attempt comprehensive measurement of condom use throughout study follow up. These studies typically ask about condom use at last sex act or, less often, over a short recent period to minimize potential for recall bias. The study will be powered to detect differences in semen exposure between the groups using this sampling. **Biomarkers provide an unbiased comparison of the frequency of semen exposure between randomized arms. In contrast, studies that rely on participant reports of exposure can be biased; the validity of reports vary by numerous study and participant factors, including HIV risk (Gallo 2011), which can result in confounding that cannot be addressed adequately in the analysis.**

We previously demonstrated in an RCT that advance knowledge of testing for PSA did not change women's behavior with respect to semen exposure or reporting of this behavior (Thomsen 2007). Thus, the act of consenting to study procedures, including sampling vaginal fluid to test for PSA, is not expected to influence participant behavior.

Preliminary data from an *in vitro* experiment conducted at the UNC laboratory provide evidence that the CSD500 condom does not interfere with the detection of PSA (Marcia Hobbs, written communication, August 2014). Laboratory technicians spiked condom extracts (including material from the inside only, outside only, or a combination of both) and non-condom controls consisting of phosphate buffered saline (PBS) only with three concentrations of PSA (0.25, 2.00 and 11.50 ng/mL) and no PSA (control). Five independent samples of each condom extract condition were prepared for a total of 60 specimens and the PBS controls. Table 1 shows the mean PSA detected for each condition, which were all within manufacturer ranges.

Table 1. Detection of PSA in PBS alone or material extracted from the inside or outside of CSD500 condoms from spiked specimens, by PSA concentrations used for spiking

Spiked PSA concentration (ng/mL)	Architect Total PSA result (mean +/- standard deviation in ng/mL) for specimens prepared with[a]			
	PBS	Condom inside	Condom outside	Condom in/out
0.00	0.00 +/- 0.00	0.00 +/- 0.00	0.00 +/- 0.00	0.00 +/- 0.00
0.25	0.23 +/- 0.01	0.23 +/- 0.01	0.22 +/- 0.01	0.22 +/- 0.01
2.00	1.88 +/- 0.03	1.88 +/- 0.08	1.89 +/- 0.07	1.95 +/- 0.12
11.50	11.74 +/- 0.29	11.76 +/- 0.59	11.71 +/- 0.59	12.08 +/- 0.31

[a]Each of the 16 test conditions (spiked PSA concentration by PBS or condom extract) evaluated for 5 independent specimens
PBS = phosphate buffered saline; PSA = prostate-specific antigen

6. STUDY POPULATION

6.1 Study site

All enrollment and data collection activities will occur at the Thanh Hoa Provincial Centre of Reproductive Health.

6.2 Eligibility criteria

The study will enroll approximately 500 couples with roughly equal distribution to the two study arms**.** Couples must speak Vietnamese to be eligible for study participation.

In addition, women must meet all of the following criteria at enrollment to be eligible:
- Be 18-45 years of age;
- Not currently using a modern contraceptive method other than condoms or intending to start using a modern contraceptive method other than condoms in the next 6 months;
- Willing to use the assigned study condoms as the sole method of contraception for the next six months;
- Not breastfeeding;
- Not known to be pregnant;
- Want to avoid pregnancy for at least the next six months; **and**
- Be in a monogamous relationship for at least the past six months with her current male partner.

Couples are ineligible for study participation if either person in the couple is known to be HIV-positive or has any of the following contraindications to CSD500 use (see Package Insert):
- History of low blood pressure or heart condition;
- Current use of medication for anemia, blood pressure, erectile dysfunction (man only), migraines, headaches or glaucoma;
- Inflamed or broken skin that the condom could come into contact with (man only); **or**
- Latex allergy or sensitivity.

Both the woman and her male partner need to be willing and able to consent to the study procedures, including the use of the assigned study condoms.

Our justification for excluding women who are known to be pregnant or already using a modern method of contraception other than condoms and couples in which either partner has a known HIV-infection is that these individuals might differ in unknown ways from others with respect to

their adherence to the promotion of condoms for avoiding semen exposure. Furthermore, individuals who are known to be HIV-positive typically receive more intensive safer sex counseling, and the scientific integrity of the study findings require that all participants receive standardized condom counseling according to their study arm.

7. STUDY AIMS

The study has three main aims.

7.1 Aim 1: To evaluate whether CSD500 provided to improve sexual pleasure, as compared to the standard condom, results in less semen exposure among women in an established relationship

We hypothesize that providing women in an established relationship with CSD500 accompanied by counseling focusing on the new condom's ability to improve male performance will result in less unprotected vaginal sex than providing the standard condom with traditional counseling focusing on disease and pregnancy prevention only. The primary outcome is the relative risk (RR) comparing PSA positivity (per established threshold of >1 ng of PSA per mL vaginal swab eluate) at the 2, 4 and 6-month follow-up visits between the two study arms.

7.2 Aim 2: To evaluate the effects of CSD500 provision on men and women's reports of sexual pleasure compared to the standard condom

We will assess sexual pleasure with the Quality of Sexual Experience (QSE) scale, which is a validated measure of the pleasure and satisfaction of event-level sexual experiences (Sanders 2013). The construct of sexual pleasure can vary dramatically between individuals. For example, when interpreting a sexual act as pleasurable, individuals could be focused on different types of activity (e.g., foreplay, touch, use of erotica or toys, and oral or anal intercourse) or different aspects of the experience (e.g., orgasm or even pain). In order to account for the diversity of experiences that might make a sexual act pleasurable for an individual, the QSE scale does not capture specific physical or emotional aspects of the experience, but rather measures seven broad, event-level, bipolar items. Note that CSD500 could increase erection duration, which could (positively or negatively) influence the man's perception of the sexual pleasure of the act. Thus, we will also consider a modified QSE scale that adds an item to assess pleasure related to the act's duration.

The QSE scale was validated for men using three single-item questions that measured the individual's assessment of 1) the overall quality of the sexual act; 2) the act compared to the individual's typical act; and 3) the occurrence and quality of the respondent's orgasm during the act (Sanders 2013). QSE scores predicted much of the variance and were statistically significantly correlated ($P < 0.001$) with each of the measures. The scale also had high internal consistency (Cronbach's alpha of 0.96).

7.3 Aim 3: To identify correlates of semen exposure measured by PSA positivity

We hypothesize that condom usage will differ by demographic (e.g., age and marital status), personal (e.g., sexual relationship power), and study-related factors (e.g., length of follow up). To date, most evidence on correlates of condom nonuse is derived from studies where women were asked to report on their sex and condom use behavior; **consequently, these studies might have identified correlates of *reporting* unprotected sex rather than correlates of**

actual unprotected sex. No published research has used a semen biomarker to characterize women in an established relationship who engage in unprotected sex.

8. STUDY PROCEDURES

For an overview, see "Schedule of study procedures."

8.1 Recruitment

Study staff will recruit women attending the Thanh Hoa Provincial Centre of Reproductive Health to the study using a standard Recruiting Script and the Study Brochure (see Attachments). Also, we will display the Study Poster (see Attachment) throughout the Thanh Hoa Provincial Centre of Reproductive Health. Initially, we will not recruit from outside of the Centre to ensure a slow rate of enrollment. Depending on the enrollment pace, we may start to recruit outside of the Centre. This recruitment will involve displaying the Study Poster and distributing the Study Brochures in places of employment (e.g., factories and school teachers) in Thanh Hoa city. This passive recruitment will require those interested in participating in the study to attend the Centre to obtain more information.

If their male partner is not present at the Centre, women will be asked to return with him to complete the enrollment visit.

Staff will complete the Eligibility Assessment Form to determine the couple's eligibility. Women who are potentially interested in starting a modern contraceptive method other than condoms will be referred following standard care and will not undergo any further recruitment to the study.

8.2 Enrollment visit

If the couple is eligible per the Eligibility Assessment Form, study staff will conduct the consent process by reading the Annotated Enrollment Consent Form aloud to the couple in a private location and answering any questions. Only couples who demonstrate comprehension of the embedded questions during the consent process and who sign the Enrollment Consent Form will be enrolled.

The REDCap software program will provide the arm assignment after each couple is enrolled into the trial. Study staff will record the couple's names, their unique participant identification numbers (PINs), and their assigned arm onto the Participant Identification Log. In the presence of the female participant only, staff will administer the Female Enrollment Questionnaire, and a study clinician will collect a double-headed vaginal swab per established procedures (Gallo 2013a). In the presence of the male participant only, staff will administer the Male Enrollment Questionnaire. Staff will provide the appropriate condom counseling (see Counseling Checklist: Control Arm and Counseling Checklist: Intervention Arm) to the couple together, and distribute the assigned study condoms. Participants in the intervention arm will receive the Futura Max Information Sheet. At the end of the enrollment visit, staff will administer the Participant Contact Form to the couple and will provide the male and female partner each with 110,000 VND (~5 USD) as compensation for their time for participation and travel costs to the clinic.

8.3 Follow-up visits

Female participants will be asked to return for follow up at 2, 4 and 6 months after enrollment. They will be instructed to return on the scheduled day regardless of their menstrual cycle. At each woman's follow-up visit, study staff will administer the Female Follow-up Questionnaire and a study clinician will collect a double-headed vaginal swab. At 2 and 4-month visits, staff will provide condom counseling (per study arm), give 20 (or more if needed based on expected coital frequency) of the

assigned study condoms, and confirm the accuracy of the information on the Participant Contact Form. At the 6-month visit, all women will receive the standard condom, which is provided for free of charge per standard care.

Men will be asked to return at the 6-month visit to complete the Male Follow-up Questionnaire.

Female participants will be compensated at the end of the 2-month (110,000 VND; ~5 USD), 4-month (220,000 VND; ~10 USD), and 6-month visits (440,000 USD; ~20 USD) for their time and travel costs. Male participants will be compensated at the end of the 6-month visit (330,000 VND; ~15 USD).

8.4 Interim visits

We will instruct participants to return to the clinic at any time for any study-related problem. Also, study staff may request that participants attend interim visits to follow-up on adverse events (AEs). All procedures at interim visits will be documented on the Adverse Event Form.

8.5 Condom counseling

Staff will provide standardized condom counseling on proper use of the assigned condom type to couples at enrollment and to the female participants at the follow-up visits (see Counseling Checklist: Control Arm and Counseling Checklist: Intervention Arm). Counseling for the two arms differs in the rationale given for condom use. Specifically, the intervention arm will receive counseling that briefly addresses condom's dual protection against pregnancy and HIV/STI but that otherwise emphasizes the potential for increased sexual pleasure with CSD500 use.

Counseling to this arm also will include CSD500-specific instructions, such as the need to briefly massage the gel inside the condom teat onto the penis head after donning the condom and to not use multiple condoms within a 24-hour period. In contrast, the control arm will receive standard counseling to use condoms for pregnancy and disease prevention without receiving any messages about the use of condoms for sexual pleasure.

Study staff will be trained with a formal curriculum (adapted from Pathfinder 2000) to counsel women on condom use. Training will address 1) key condom messages (per the intervention arm as described above); 2) mechanism of action and condom effectiveness; 3) factors that influence condom effectiveness; 4) indications and rationale for condom use; 5) counseling patients on correct condom use; and 6) correct condom storage procedures in service delivery settings. Only staff who demonstrate proficiency will provide condom counseling to study participants.

8.6 Specimen collection and testing

Trained clinicians will collect a vaginal fluid specimen (using a 1-mL, rayon-tipped double-headed swab) from each participant at each of the four scheduled study visits. Swabs will be stored onsite at −70°C following collection until their shipment in batches on dry ice to the UNC laboratory, where one of the double-headed swabs will be processed and tested in batches for total PSA (Architect Total PSA; Abbott Diagnostics, Abbott Park, IL) by trained laboratory personnel following established procedures (Gallo 2013a). The second of the double-headed swabs may be used for quality assurance testing during the study.

8.7 Referrals

We will follow standard of care (MOH 2002) to refer participants for HIV/STI screening and care at the site. This care is not part of the study.

8.8 Contact tracing

Study staff will attempt to trace participants – using contact information provided by the participant for this purpose – if participants do not return for their Follow-up Visits in a timely manner.

8.9 Early study discontinuation

Participants have the right to withdraw their participation from the study at any time for any reason. They may also be discontinued if the study staff determines that further participation would be detrimental to the participants' health or well-being. All incidences of study discontinuation will be documented on the Study Discontinuation Form.

9. STATISTICAL CONSIDERATIONS

9.1 Sample size

Figure 2. Samples sizes per arm at various levels of rho for powers of 0.8 and 0.9 to detect RR of 0.5 to 0.6*

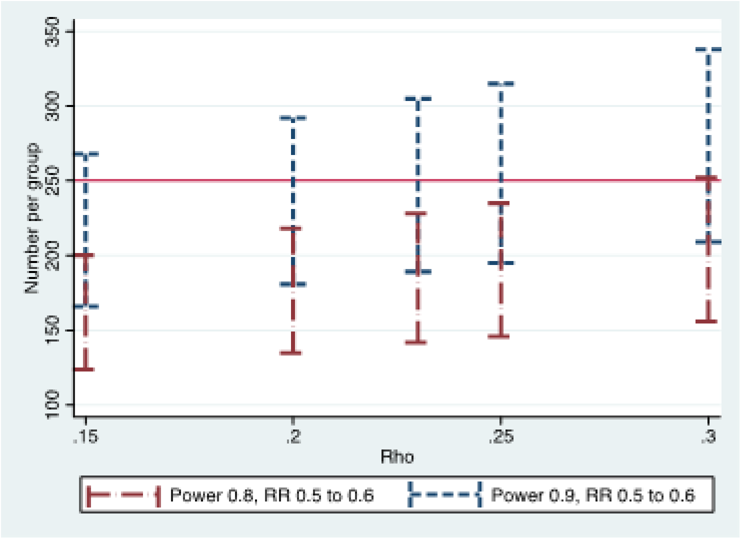

****Alpha = 0.05, PSA positivity in control arm = 0.15, attrition = 0.1 of participants fail to return post-enrollment and 0.25 of the remainder miss one follow-up visit)***

Sample size calculations are based on detecting a clinically relevant RR of PSA positivity between the intervention and control arms. Our primary outcome is PSA positivity at the three follow-up points for each participant. Sample size calculations are based on formulae for correlated binary response variables (Fitzmaurice 1995; Jung 2003), using specifically GEE to fit a log-binomial model (Rochon 1998; Dahmen 2004). We show calculations (Figure 1) based on significance level of 0.05 (two-sided test), power to detect the effect set at 80% and 90%, and probability of PSA positivity in the control arm of 0.15. While the frequency of PSA positivity observed in past research has ranged from 8-38% (Table 2), these studies almost uniformly either studied populations with PSA positivity that could be expected to be higher (sex workers) or lower (syndromic STI patients or women in serodiscordant relationships) than in the proposed trial. Only two trials studied HIV-negative women who were not sex workers or syndromic STI patients; PSA positivity in these trials ranged from 0.22 to 0.25 (Minnis 2009). To be conservative in ensuring an adequate sample size, we reduced our expected frequency of positivity roughly by 1/3 to 0.15. We assume an exchangeable correlation structure (same correlation, rho, between any two observations on the same subject). Given that previous studies produced an estimated rho of approximately 0.23 (Anderson 2013; Hobbs 2009; Gallo 2007), we display sample sizes required for values of rho ranging from 0.15 to 0.3. We considered RRs from 0.5 to 0.6, meaning a reduction of PSA positivity from 15% in the control arm to between 7.5% and 9% in the intervention arm. Finally, we adjusted the sample

Table 2. Frequency of observed PSA positivity, by study

Study	Location	Female population	PSA tests	PSA positivity No.	PSA positivity (%)
Hobbs 2003	Bangladesh	Sex workers	402	154	(38.3)
Evans 2013	Cambodia	Sex workers	183 at month 6<br>172 at month 9<br>170 at month 12	42<br>42<br>33	(23.0)<br>(24.4)<br>(19.4)
Pépin 2006	Guinea, Togo, Ghana & Mali	Sex worker, syndromic STI patients<br>Syndromic STI clinic patients	508<br>658	94<br>58	(18.5)<br>(8.8)
Aho 2010	Guinea	Sex workers	219	84	(38.4)
Anderson 2013	Jamaica	Syndromic STI patients	285 at day 0<br>286 at day 6	24<br>29	(8.4)<br>(10.1)
**Rattray [in press][a]**	**Jamaica**	**HIV-negative, seeking contraception**	**414 at month 0<br>401 at month 1<br>389 at month 3**	**103<br>93<br>95**	**(24.9)<br>(23.2)<br>(24.4)**
Gallo 2007	Kenya	Sex workers	194 at month 0<br>183 at month 12	30<br>21	(15.5)<br>(11.8)
Mose 2013	Kenya	In a HIV-discordant relationship	124	3	(10.5)
Gallo 2006	Madagascar	Sex workers	332	126	(38.0)
**Minnis 2009[a]**	**Zimbabwe**	**Sexually-active, HIV-negative**	**910**	**196**	**(21.5)**

[a]*Two trials of HIV-negative women who were not sex workers or syndromic STI patients*

sizes to account for attrition. Specifically, we assumed that 10% would fail to return after enrollment and, among the 90% remaining in the study, the probability of missing a follow-up visit would be 25%.

Given these assumptions, 500 subjects split equally in two arms will provide us with adequate power (80% for all assumed ranges of values and 90% in many) to detect a clinically meaningful reduction in PSA positivity for most values of rho. For example, at the estimated rho of 0.23, this sample size yields 80% power to detect a RR of approximately 0.61 at alpha level of 0.05. Thus, a total sample of 500 women will ensure adequate power to detect a clinically meaningful change in the frequency of PSA positivity between the study arms.

9.2 Randomization

After having been determined to be eligible and having provided written consent for participation, couples will be randomized to one of the two study arms in roughly equal proportions. The Statistician will prepare the randomization program in REDCap before study initiation. The concealment of allocation process will be accomplished using the relevant function in the REDCap electronic data capture system.

9.3 Data analysis

The success of the study objectives will be judged by results of statistical tests. Each objective will be operationalized in three steps: 1) specific endpoints are identified and measured; 2) statistical hypotheses relate the objectives to the measured endpoints; and, 3) the statistical

hypotheses are tested, using appropriate analytic procedures. Supportive analyses will include simple descriptive tables at baseline. The frequency and causes of missing and mistimed data values will be examined.

An intent-to-treat analysis will be used for the primary analysis in Aim 1. To that end, every effort will be made to obtain complete data on participants, including those who report not using (or failing to successfully use for the entire act) the assigned condoms. All variable data values will be used whenever possible.

The following sections describe the plans for formal estimation and inference.

9.3.1 Analysis of Aim 1

We hypothesize that the frequency of PSA detection (defined as <1 ng/mL) during the six months of follow up will be lower in the intervention arm compared to the control arm. To test our hypothesis, we will calculate a relative risk (RR) with 95% confidence interval (CI) for the frequency of PSA positivity between arms, using generalized estimating equation (GEE) methods to account for correlation across visits from individual participants. Sensitivity analyses will include 1) calculating the RR of PSA positivity at least once during follow up (i.e., using women, instead of visits, as the unit of analysis); 2) using a higher threshold (4 ng/mL) to define PSA positivity; and 3) considering the effect of study duration on the main comparison. These analyses will be based on an intent-to-treat design (Lachin 2000). Although masking participants and clinic study staff to arm assignment is not feasible, the Statistician and laboratory staff will remain masked until the primary analyses are completed. We will follow CONSORT guidelines for reporting the primary results (Moher 2001).

9.3.2 Analysis of Aim 2

We hypothesize that couples randomized to receive CSD500 will experience greater sexual pleasure compared to those receiving the standard condom. To test our hypothesis, we will use linear regression to compare QSE scores for the most recent use of a study condom for vaginal intercourse between women in the intervention and control arms at the 2, 4 and 6-month visits using GEE methods and between men in the two arms at the 6-month visit. We will repeat these analyses using an alternative measure of sexual pleasure, the Sexual Experience Questionnaire (SEX-Q) (Jones et al. 2008). Other secondary analysis will include 1) evaluating whether PSA positivity (defined as <1 ng/mL) is associated with women's QSE scores, women's perceptions of their partner's pleasure, men's QSE scores, and men's SEX-Q scores (in separate models) and 2) men's acceptability of the study condom using descriptive statistics. In the event that the intervention is not statistically associated with a reduction in observed PSA positivity (i.e., the hypothesis for Aim 1 is not supported), these data could be useful for elucidating relationships between sexual pleasure and condom use within specific subgroups of the study population.

9.3.3 Analysis of Aim 3

We will evaluate the following as potential correlates of semen exposure (PSA positivity defined as >1 ng/mL) at enrollment and the three follow-up visits: 1) woman's sexual relationship power, as measured by the sexual relationship power scale (SRPS) (Pulerwitz 2000); 2) her or her partner's regulatory focus (promotion versus prevention-focus) (Gomez 2013); 3) her partner's perceived barriers to condom use, as measured by the Condom Barriers Scale (St. Lawrence 1999), which has been modified for use in males (Doyle 2009); 4) variables based on the Theory of Gender and Power as applied to women's HIV risk (e.g., age, difference in age with

her male partner, prior condom experience, past sexual partners, history of other contraceptive method use, alcohol or other drug use, and perceived risk of infection or pregnancy and degree of motivation to avoid these) (Wingood 2000). Secondary analyses will include 1) identifying correlates of self-reported recent semen exposure (dichotomous variable for any report of vaginal sex without condom use with study partner in the past 48 hours) and 2) assessing whether correlates of semen exposure differ according to the outcome measure (PSA positivity or self-report). To conduct the latter exploratory analysis, we will simultaneously fit models for the two outcomes using bivariate logistic regression (Fitzmaurice 1995). We will apply GEE with alternating logistic regression (Carey 1993), which directly models within-woman associations using pairwise odds ratios. This bivariate approach will allow us to directly test whether the two models differ rather than assume that a significant finding in one model but not the other would imply that the two outcomes differ in their risk factors.

10. DATA MANAGEMENT

Given the potential for low participant literacy, trained study staff will administer all study forms and questionnaires and will electronically record responses directly onto a laptop computer using REDCap software. REDCap, which is supported by the Center for Clinical and Translational Science (CCTS) at OSU, facilitates the transmittal of secure, encrypted and password-protected data. CCTS will prepare the data entry screens with built-in quality control checks. Entered data will be instantaneously uploaded behind the secure OSU firewall. The PI will monitor the data during the study to resolve any potential issues upon detection.

In the case of internet outage or other technical difficulties, study staff will record data onto paper forms using a pen. These data will be entered into REDCap when the internet access is restored. PSA test results will be sent from the UNC laboratory to the PI in an electronic spreadsheet. The Site Investigator and PI will review the data files on an ongoing basis for accuracy. To maintain participant confidentiality, only a coded PIN will identify all data collection forms (DCFs), reports, and other records. Participants' names will be stored separately in a locked file in a locked office at the study site. No reports will link individual names with person-level data. Unique PINs will be used to link participant-specific data. All records will be secured in a locked room, accessible only by trained study staff. All computer entry and networking programs will be done with coded numbers only. Electronic files will be password-protected, with access only by authorized study personnel. Clinical information will not be released without written permission of the participant, except as necessary for monitoring by OSU or its designee.

11. TRANSLATION

All study materials will be translated into Vietnamese. After translation into Vietnamese, the DCF, Enrollment Consent Form and Futura Max Information Sheet will be back translated into English to ensure linguistic equivalence between the two versions.

12. INCIDENTS AND INCIDENT REPORTING

12.1 Adverse events

Adverse events (AEs) are defined as any untoward health-related reaction, effect, toxicity, or abnormal laboratory result that a study participant experiences during the course of a study. Only new untoward health-related events or worsening of a baseline condition are considered AEs.

Study staff will record information on all AEs that are classified 1) as possibly, probably, or definitely related to study participation **and** as unexpected or 2) as serious. An *unexpected* AE is one whose specificity or severity is not listed in the CSD500 package insert. Expected AEs include headaches, faintness and nausea, loss of sensation, dizziness and skin irritation. Information on expected AEs will be recorded on study DCFs, but these events will not be reported to the relevant IRBs unless the event is also serious or is an unanticipated problem involving risks to subjects or others (UPIRTSO).

An AE is considered *serious* if it is fatal or life-threatening, requires in-patient hospitalization or prolongation of an existing hospitalization, results in a persistent or significant disability or incapacity, is a congenital anomaly or birth defect, jeopardizes the participant or requires intervention to prevent one of the outcomes listed above, or any other event deemed serious by the investigator. Study personnel will report any AE to the Site Investigator, who will determine 1) its relatedness to study participation and 2) its seriousness.

All SAEs will be reported in an expedited manner by completing the Adverse Event Form within two working days of when the site identifies the SAE. If it is determined that the SAE is unanticipated and anything other than “not related” (i.e., possibly, probably or definitely related), then the Site Investigator and PI will coordinate the notification of the relevant IRBs.

Non-serious AEs will be recorded on DCFs but do not require expedited reporting unless they are classified as UPIRTSO. All AEs will be managed according to the standard of care for the clinical practice and the judgment of the on-site clinicians.

12.2 Reporting other incidents that are unanticipated problems involving risks to subjects or others (UPIRTSO) incidents

UPIRTSO are any events or information that were unexpected and indicate that research procedures caused harm to participants or others or indicate that participants or others are at increased risk of harm. An unanticipated problem may involve any aspect of the research study and could involve anyone including participants, research staff, or others not directly related to the study. AEs that are unexpected and related and protocol violations are specific examples of UPIRTSO. Not all AEs are UPIRTSO, and not all UPIRTSO are AEs.

Examples of other UPIRTSO include 1) a breach of confidentiality; 2) a change to the protocol taken without prior IRB review to eliminate an apparent immediate hazard to a research participant; 3) halting of the study by a sponsoring agency; and 4) a participant complaint that indicates unexpected risks or that cannot be resolved by the study team. Any possible UPIRTSO will be recorded on the appropriate log and report form. The Site Investigator or designee will decide whether the incident meets the definition of UPIRTSO and, thus, requires prompt reporting to the IRBs.

12.3 Sponsor and principal investigator responsibilities

The sponsors, PI, Site Investigator, or designees have the authority to stop the study due to SAEs, UPIRTSOs, protocol violations, or problems related to inadequate recruitment or follow- up, or upon the direction of an IRB.

The Site Investigator or designee has the authority to change the protocol to eliminate an apparent immediate hazard to a research participant.

13. PROTECTION OF HUMAN SUBJECTS

13.1 Informed consent process

Study staff will read the Annotated Enrollment Consent Form aloud to the couple. This Form is identical to the Enrollment Consent Form except that the former has questions embedded to ask to the potential participants to gauge their comprehension of the study during the consent process. Only couples who demonstrate comprehension of the answers to these questions will be enrolled.

Both members of the couple will indicate their agreement to participate in the study by signing the Enrollment Consent Form. Individuals who are illiterate will provide an “X” on the form to indicate consent; the full discussion of the consent process for these participants will be conducted in the presence of a witness, who will sign the Enrollment Consent Form indicating that the witness has observed the entire consent process. The female and male participants will be asked for their consent for future contact about new research. They will indicate whether they consent to this by checking the appropriate box on the Enrollment Consent Form. The couple do not consent to future contact, they can still enroll in the study. The couple will be given an unsigned copy of the Enrollment Consent Form to keep for future reference, as it provides contact information for staff responsible for the conduct of the research study in the event of any questions or concerns.

13.2 Minimizing coercion or undue influence

The reimbursements for participation have been set in line with the local economy and recent research studies conducted with this population and will only compensate participants for their time in completing the questionnaires. The Enrollment Consent Form specifies the reimbursement amounts in the local currency, VND.

13.3 Description of risks

This study poses no more than minimal risk to participants. The active ingredient in CSD500 can cause headaches, faintness and nausea, loss of sensation, dizziness and skin irritation (see Package Insert). Participants will be instructed to stop using the study condoms if either they or their partner experience any of these symptoms and will be referred for standard care, as indicated.

Participants may experience psychological risks associated with answering questions about sensitive topics or reporting attitudes and behaviors, such as their sexual activity and details of their sexual relationships. Several measures have been implemented to minimize these risks. First, during the consent process, participants will be advised that they are free to decline study participation. Participants also will be informed that they can decline to answer any questions. Participants also will be informed of their freedom to terminate the interview or visit at any time. All interviews that take place in-person will occur in a private setting by interviewers who have been trained thoroughly on the importance of confidentiality, the procedures to protect a participant’s confidentiality, and the penalties associated with breaches of confidentiality.

Participants' personal information will be kept confidential. Access to the signed consent forms and the Participant Identification Log (i.e., the roster linking participant names and PINs) will be limited to authorized study personnel and remained locked in a file cabinet when not in use.

We will not perform any genetic testing.

13.4 Description of anticipated benefits to the research participant

There are no study benefits to the research participant. Participants and others may indirectly benefit in the future from the trial results.

13.5 Description of potential risk to anticipated benefit ratio

The potential risks involved in trial participation are reasonable in relation to the anticipated benefits.

13.6 Provisions for protecting privacy and confidentiality

Participants' privacy and the confidentiality of data will be protected through training of interviewers and other study staff; conducting all interviewing, and physical examinations in private; storing study materials in a locked room; and securing computer files that include identifiers. Only the study PINs will identify participant research records. Linkages between PINs and participants' identifying information will not be maintained in a computer database; rather, they will be maintained in a paper log that is kept locked and only accessible to study staff at the study site. These linkages will be destroyed at an agreed upon timeframe after the main study articles are published. Data identifying individual study participants will not be published or released to persons outside of the project.

13.7 Treatment and compensation for injury

No money has been set aside for compensation for injury from study participation; however, this study poses no more than minimal risk to participants.

13.8 Data and Safety Monitoring Committee (DSMC)

The Regulatory and Ethics Core of the CCTS at OSU will assemble a Data Safety Monitoring Committee (DSMC) – consisting at minimum of a research ethicist / research subject advocate, biostatistician, and gynecologist – to independently monitor the safety of the study. The DSMC will review 1) the protocol before the accrual of human subjects; and the accumulated data after 2) approximately 50-100 women-months of follow up have been completed; 3) after approximately half of the expected women-months have been accrued; and 4) after study follow-up has ended. The DSMC will evaluate participant safety and study conduct and progress and will make recommendations to the sponsor and investigators concerning the trial continuation, modification or termination. No specific stopping guidelines will be presented with the data, and recommendations for stopping early for safety reasons will be based on the judgment of the DSMC. The Committee will approve a detailed DSMC plan before the accrual of human subjects.

14. PLANS FOR REPORTING RESULTS

Upon completion of data analyses, the Site Investigator will organize the dissemination of the main study findings to the study team in Vietnam as well as to local stakeholders. In addition, the primary results will be written up for publication in a peer-reviewed scientific journal. Additional papers will also be written and submitted to peer-reviewed scientific journals as appropriate.

A publication policy will be established to govern the publication of the results of the trial. Authorship roles will be assigned primarily according to the contributions of individual members as well as the likelihood that the authors will be able to complete data analysis and preparation of manuscripts in a timely manner. All investigators will be encouraged to participate in the discussions regarding authorship. All abstracts and publications resulting from this study must be cleared and approved by all participating institutions prior to submission to scientific meetings or journals.

15. REFERENCES

Aho J, Koushik A, Diakité SL, et al. Biological validation of self-reported condom use among sex workers in Guinea. AIDS Behav 2010;14:1287-93.

Anderson C, Gallo MF, Hylton-Kong T, et al. Randomized controlled trial on the effectiveness of counseling messages for avoiding unprotected sexual intercourse during STI and RTI treatment among female patients. Sex Transm Dis 2013;40:105-10.

Carey V, Zeger SL, Diggle P. Modelling multivariate binary data with alternating logistic regressions. Biometrika 1993;80:517-26.

Cecil M, Nelson A, Trussell J, et al. If the condom doesn’t fit, you must resize it. Contraception 2010;82:89-90.

Cooper TG, Noonan E, von Eckardstein S, et al. World Health Organization reference values for human semen characteristics. Human Reprod Update 2010;16:231-45.

Crosby RA, Milhausen RR, Mark KP, et al. Understanding problems with condom fit and feel: an important opportunity for improving clinic-based safer sex programs. J Prim Prev 2013;34:109-15.

Crosby RA, Yarber WL, Graham CA, et al. Does it fit okay? Problems with condom use as a function of self-reported poor fit. Sex Transm Infect 2010;86:36-8.

Dahmen G, Rochon J, König IR, et al. Sample size calculations for controlled clinical trials using generalized estimating equations (GEE). Methods Inf Med 2004;43:451-6.

Daniels K, Daugherty J, Jones J. Current contraceptive status among women aged 15–44: United States, 2011–2013. NCHS data brief, no 173. Hyattsville, MD: National Center for Health Statistics. 2014.

Do M, Fu H. Is women's self-efficacy in negotiating sexual decisionmaking associated with condom use in marital relationships in Vietnam? Stud Fam Plann 2011;42:273-82.

Doyle SR, Calsyn DA, Ball SA. Factor structure of the Condoms Barriers Scale with a sample of men at high risk for HIV. Assessment. 2009;16:3-15.

Evans JL, Couture MC, Stein ES, et al. Biomarker validation of recent unprotected sexual intercourse in a prospective study of young women engaged in sex work in Phnom Penh, Cambodia. Sex Transm Dis 2013;40:462-8.

Fitzmaurice GM, Laird NM, Zahner GEP, et al. Bivariate logistic regression analysis of childhood psychopathology rating using multiple informants. Am J Epidemiol 1995;142:1194-203.

García SG, Goldman L. Understanding successful condom use in the Dominican Republic: FRONTIERS final report. Washington: Population Council, 2004.

García SG, Goldman L. Understanding successful condom use in northern Mexico: final narrative report. New York: Population Council, 2003.

Gallo MF, Behets FM, Steiner MJ, et al. Prostate-specific antigen to ascertain reliability of self-reported coital exposure to semen. Sex Transm Dis 2006;33:476-9.
Gallo MF, Behets FM, Steiner MJ, et al. Validity of self-reported “safe sex” among female sex workers in Mombasa, Kenya – PSA analysis. Int J STD AIDS 2007;18:33-8.
Gallo MF, Snead MC, Black CM, et al. Optimal methods for collecting and storing vaginal specimens for prostate-specific antigen testing in research studies. Contraception 2013a;87:830-5.
Gallo MF, Sobel JD, Rompalo AM, et al. Discordance between spermatozoa detection and self-reported semen exposure. Sex Transm Dis 2011;38:909-12.
Gallo MF, Steiner MJ, Hobbs MM, et al. Biological markers of sexual activity: tools for improving measurement in HIV/STI prevention research. Sex Transm Dis 2013b;40:447-52.
Gomez P, Borges A, Pechmann C. Avoiding poor health or approaching good health: Does it matter? The conceptualization, measurement, and consequences of health regulatory focus J Consum Psychol 2013;23:451-63.
Graham CA, Crosby R, Yarber WL, et al. Erection loss in association with condom use among young men attending a public STI clinic: potential correlates and implications for risk behaviour. Sex Health 2006;3:255-60.
Graves HC, Sensabaugh GF, Blake ET. Postcoital detection of a male-specific semen protein. Application to the investigation of rape. N Engl J Med 1985; 312:338Y343.
Hensel DJ, Stupiansky NW, Herbenick D, et al. Sexual pleasure during condom-protected vaginal sex among heterosexual men. J Sex Med 2012;9:1272-6.
Higgins JA, Hirsch JS. Pleasure, power, and inequality: incorporating sexuality into research on contraceptive use. Am J Public Health 2008;98:1803-13.
Higgins JA, Tanner AE, Janssen E. Arousal loss related to safer sex and risk of pregnancy: Implications for women’s and men’s sexual health. Perspect Sex Reprod Health 2009;41:150-7.
Hobbs MM, Steiner MJ, Rich KD, et al. Good performance of rapid prostate-specific antigen test for detection of semen exposure in women: implications for qualitative research. Sex Trans Dis 2009;36:501-6.
Holmes KK, Levine R, Weaver M. Effectiveness of condoms in preventing sexually transmitted infections. Bull World Health Organ 2004;82:454-61.
Jamshidi R, Penman-Aguilar P, Macaluso M, et al. Detection of two biological markers of intercourse: prostate-specific antigen and Y-chromosomal DNA. Contraception 2013;88:749-57.
Jones LA, Klimberg IW, McMurray JG, et al. Effect of sildenafil citrate on the male sexual experience assessed with the Sexual Experience Questionnaire: a multicenter, double-blind, placebo-controlled trial with open-label extension. J Sex Med 2008;5:1955-64.
Jung SH, Ahn C. Sample size estimation for GEE method for comparing slopes in repeated measurements data. Stat Med 2003;22:1305-16.
Kamenev L, Leclercq M, Francois-Gerard C. An enzyme immunoassay for prostate-specific p30 antigen detection in the postcoital vaginal tract. J Forensic Sci Soc 1989;29:233-41. Lachin JM. Statistical considerations in the intent-to-treat principle. Control Clin Trials 2000;21:167-89.
Lawson ML, Macaluso M, Bloom A, et al. Objective markers of condom failure. Sex Transm Dis 1998;25:427-32.
Lin J, Whitlock E, O'Connor E, Bauer V. Behavioral counseling to prevent sexually transmitted infections. Rockville, MD: U.S. Agency for Healthcare Research and Quality; 2008. Report No.: 08-05123-EF-1. U.S. Preventive Services Task Force Evidence Syntheses.
Macaluso M, Lawson L, Akers R, et al. Prostate-specific antigen in vaginal fluid as a biologic marker of condom failure. Contraception 1999;59:195-201.

Mehryar A. Condoms: awareness, attitudes and use. In: Cleland J, Ferry B, eds. Sexual Behaviour and AIDS in the Developing World. London: Taylor and Francis, 1995.
Ministry of Health of Vietnam. Survey Assessment of Vietnamese Youth (SAVY). Hanoi: Ministry of Health of Vietnam, General Statistics Office of Vietnam, United Nations Children's Fund, World Health Organization, 2005.
Ministry of Health of Vietnam. The National Strategy for Reproductive Health for the 2001-2010 Period. Hanoi: MOH, 2002.
Ministry of Health of Vietnam. Vietnam HIV/AIDS estimates and projections 2007–2012. Hanoi: Family Health International, 2009.
Minnis AM, Steiner MJ, Gallo MF, et al. Biomarker validation of reports of recent sexual activity: results of a randomized controlled study in Zimbabwe. Am J Epidemiol 2009;170:918-24.
Moher D, Schulz KF, Altman DG. The CONSORT statement: revised recommendations for improving the quality of reports of parallel-group randomised trials. Lancet 2001;357:1191-4.
Moreno R, Nababan HY, Ota E, et al. Structural and community level interventions forincreasing condom use to prevent the transmission of HIV and other sexually transmitted infections. Cochrane Database Syst Rev 2014;7:CD003363.
Mose F, Newman LP, Njunguna R, et al. Biomarker evaluation of self-reported condom use among women in HIV-discordant couples. Int J STD AIDS 2013;24:537-40.
Nguyen TA, Oosterhoff P, Hardon A, et al. A hidden HIV epidemic among women in Vietnam. BMC Public Health 2008;8:37.
O'Leary A. Are dual-method messages undermining STI/HIV prevention? Infect Dis Obstet Gynecol 2011;2011:691210.
Pathfinder International. Women Comprehensive Reproductive Health and Family Planning Training Curriculum. Module 9: Condoms and Spermicides. Watertown, MA: Pathfinder International, 2000. Available at http://www2.pathfinder.org/pf/pubs/module9.pdf
Pépin J, Fink GD, Khonde N, et al. Improving second-generation surveillance: the biological measure of unprotected intercourse using prostate-specific antigen in vaginal secretions of West African women. J Acquir Immune Defic Syndr 2006;42:490-3.
Philpott A, Knerr W, Maher D. Promoting protection and pleasure: amplifying the effectiveness of barriers against sexually transmitted infections and pregnancy. Lancet 2006;368:2028-31.
Pulerwitz J, Gortmaker SL, De Jong W. Measuring relationship power in HIV/STD research. Sex Roles 2000;42:637-60.
Randolph M, Pinkerton S, Bogart L, et al. Sexual pleasure and condom use. Arch Sex Behav 2007;36:844-8.
Rattray C, Wiener J, Legardy-Williams J, et al. Effect of initiating a contraceptive implant on subsequent condom use: a randomized controlled trial. Contraception [In press].
Rochon J. Application of GEE procedures for sample size calculations in repeated measures. Stat Med 1998;17:1643-58.
Sanders SA, Herbenick D, Reece M, , et al. The development and validation of a brief Quality of Sexual Experience (QSE) scale: results from a nationally representative sample of men and women in the United States. J Sex Med 2013;10:2409-17.
Sanders SA, Hill BJ, Crosby RA, et al. Correlates of condom-associated erection problems in young, heterosexual men: condom fit, self-efficacy, perceptions, and motivations. AIDS Behav 2014;18:128-34.
Sanders SA, Yarber WL, Kaufman EL, et al. Condom use errors and problems: a global view. Sex Health 2012;9:81-95.
St Lawrence JS, Chapdelaine AP, Devieux JG, et al. Measuring perceived barriers to condom use: psychometric evaluation of the Condom Barriers Scale. Assessment 1999;6:391-404.
Thomsen SC, Gallo MF, Ombidi W, et al. Randomized controlled trial on whether advance knowledge of prostate-specific antigen testing improves participant reporting of unprotected sex. Sex Transm Infect 2007;83:419-20.

Trussell J. Contraceptive failure in the United States. Contraception 2011;83:397-404.
UNAIDS. HIV transmission from men to women in intimate partner relationships in Viet Nam: a Discussion Paper. UNAIDS, 2010. Accessed on September 23, 2014 at http://www.un.org.vn/en/publications/un-widepublications/doc_details/221-hiv-transmission-from-men-to-women-in-intimate-partner-relationships-invietnam-a-discussion-paper.html
Weller S, Davis K. Condom effectiveness in reducing heterosexual HIV transmission. Cochrane Database Syst Rev 2002;(1):CD003255.
Wingood GM, DiClemente RJ. Application of the theory of gender and power to examine HIV-related exposures, risk factors, and effective interventions for women. Health Educ Behav. 2000;27:539-65.

16. ATTACHMENTS

1. Study timeline

2. Schedule of study procedures

3. Futura Max package insert

4. Futura Max packaging design

5. Recruitment Script

6. Counseling Checklist
    - Control Arm
    - Intervention Arm

7. Futura Max Information Sheet

8. Letters of support
    - Luong Ngoc Truong, MD
    - Luu Thi Hong, MD, PhD
    - David Davies

9. Data Safety and Monitoring Committee Charter

10. Data collection forms
    - Eligibility Assessment Form
    - Randomization Assignment Form
    - Vaginal Swab Collection Form
    - Female Enrollment Questionnaire
    - Male Enrollment Questionnaire
    - Female Follow-up Questionnaire
    - Male Follow-up Questionnaire
    - Study Discontinuation Form
    - Interim Visit Form
    - Adverse Event Form

11. Enrollment Consent Form

12. Annotated Enrollment Consent Form

Study timeline

	Year 1				Year 2				Year 3				Year 4				Year 5			
**Quarter:**	**1**	**2**	**3**	**4**	**1**	**2**	**3**	**4**	**1**	**2**	**3**	**4**	**1**	**2**	**3**	**4**	**1**	**2**	**3**	**4**
If relevant, obtain IRB approvals on protocol amendment	■	■																		
Form Data Safety Monitoring Committee	■																			
Pilot counseling messages and data collection forms	■																			
Create study manual of operation including logs and forms		■	■	■																
Finalize data management plan			■	■	■															
Test and finalize electronic versions of data collection forms			■	■																
Hire study staff				■	■															
Develop training curricula				■																
Conduct site pre-initiation visit, staff training, and “wet” run					■															
Conduct recruitment, enrollment, and follow up					■	■	■	■	■	■	■	■	■	■	■	■	■			
Clean data					■	■	■	■	■	■	■	■	■	■	■	■	■			
Conduct data analysis																		■	■	■
Disseminate study findings																		■	■	■

Schedule of study procedures

Study procedure	Visit				
	Enroll-ment	2-month	4-month	6-month	Interim
Participant identification number (PIN) assigned	⚤				
Eligibility Assessment Form	⚤				
Enrollment Consent Form	⚤				
Randomization Assignment Form	⚤				
Female Enrollment Questionnaire	♀				
Male Enrollment Questionnaire	♂				
Condom counseling	⚤[a]	♀[a]	♀[a]	⚤	
Condom provision	⚤[a]	♀[a]	♀[a]	♀	†
Provide Futura Max Information Sheet	⚤[a]	♀[a]	♀[a]		
Vaginal swab collection and Form	♀	♀	♀	♀	
Female Follow-up Questionnaire		♀	♀	♀	
Male Follow-up Questionnaire				♂	
Study Discontinuation Form	†	†	†	†	†
Interim Visit Form					†
Adverse Event Form	†	†	†	†	†
Obtain contact information	⚤	♀	♀		
Participant compensation	⚤	♀	♀	⚤	

Procedure completed by couple (⚤); woman only (♀); man only (♂); only if needed (†)
[a]According to randomized arm assignment

VN CSD500 Study
Counseling Checklist: Control Arm

1.	Greets patient respectfully.<br>• Assures privacy.<br>• Tries to put at ease if shy or embarrassed.	☐
2.	Asks what patient knows about condoms and about past experience with condoms.<br>• Corrects any myths or incorrect information.	☐
3.	Provides basic facts about condoms.<br>• Explains that condoms provide a barrier to 1) protect man and woman against spread of HIV and other sexually transmitted infections and 2) woman against pregnancy.<br>• Counsels talking with partner about using condoms.<br>• Asks if has any questions and answers them.	☐
4.	Provides specific instruction on how to correctly use and when to use condoms.<br>• Couple should use during every act of intercourse.<br>• Couple should not "test" condoms by blowing up or unrolling.<br>• Condom should be put on when penis is erect.<br>• Condom should be put on before the penis comes near or is put into the vagina.	☐
5.	**Demonstrates** how to correctly put on condom by using a model, banana, or two fingers.<br>• Cautions **not to unroll** condom before putting on.<br>• Shows how to place condom rim on penis and how to unroll up to penis base.<br>• Instructs on how to leave 1/2 inch space for semen at tip of condom, which must not be filled with air or it may burst.<br>• Shows how to expel air by pinching tip of condom as it is put on.<br>• Cautions about tearing accidentally with fingernails or rings.	☐
6.	Counsels about what to do if condom breaks or slips off during sex.<br>• Woman should return to clinic to be assessed for emergency contraception.<br>• Woman should request emergency contraceptive pills within 72 hours (the earlier the better) of unprotected sex or breakage of a condom.	☐
7.	Has patient demonstrate and practice putting on condom using the model, banana or fingers.<br>• Corrects any technique errors.	☐
8.	Counsels patient on how to remove penis from vagina with condom intact and without spilling semen.<br>• Hold on to rim of condom when withdrawing.	☐

	• Be careful not to let semen spill into vagina when penis is flaccid.	
9.	Discusses use of lubricants and what not to use.<br>• Do not use any of these: petroleum-based products (e.g., Vaseline); mineral, vegetable, or cooking oil; baby oil; margarine or butter.	☐
10.	Advises to use a spermicide or water-based lubricant if one is needed.	☐
11.	Advises patient to dispose of condoms by burning, burying, or throwing in the latrine.	☐
12.	Repeats main condom messages to patient.<br>• Be sure to have condom on hand before you need it.<br>• Use a condom with every act of intercourse.<br>• Do not use a single condom more than once.<br>• Do not rely on condom if package is damaged, torn, outdated, dry, brittle or sticky.	☐
13.	Provides patient with condoms for two months.<br>• Multiply patient's expected weekly coital frequency by 9. Give her this number of condoms or, if this number is less than 20, give her 20 condoms.	☐
14.	Instructs patient not to share study condoms with anyone but main partner.<br>• Do not share study condoms with family or friends.	☐
15.	Reassures patient that should return at any time for advice or more condoms.	☐

VN CSD500 Study
Counseling Checklist: Intervention Arm

1.	Greets patient respectfully.<br>• Assures privacy.<br>• Tries to put at ease if shy or embarrassed.	☐
2.	Asks what patient knows about condoms and about past experience with condoms.<br>• Corrects any myths or incorrect information.	☐
3.	Provides basic facts about condoms.<br>• Briefly mentions that condoms protect against pregnancy and HIV and other sexually transmitted infections.<br>• **Explains that patient is receiving the new Futura Max condom. Futura Max condoms have a small amount of a special gel inside of the condom. This gel increases blood flow within the man's penis. This makes the man's erection harder and last longer. This can make sex an intense and more pleasurable experience for the couple.**<br>• Counsels talking with partner about using Futura Max condoms.<br>• Asks if has any questions and answers them.	☐
4.	Provides specific instruction on how to correctly use and when to use condoms.<br>• Couple should use during every act of intercourse.<br>• Couple should not "test" condoms by blowing up or unrolling.<br>• Condom should be put on when penis is erect.<br>• Condom should be put on before the penis comes near or is put into the vagina.<br>• Couple should not use more than one Futura Max condom within 24 hours. If having sex again within 24 hours, use a standard condom.	☐
5.	**Demonstrates** how to correctly put on condom by using a model, banana, or two fingers.<br>• Cautions **not to unroll** condom before putting on.<br>• Shows how to place condom rim on penis and how to unroll up to penis base.<br>• Instructs on how to leave 1/2 inch space for semen at tip of condom, which must not be filled with air or it may burst.<br>• Shows how to expel air by pinching tip of condom as it is put on.<br>• **Explains that once the condom is on the penis, the man or woman should squeeze the teat of the condom and massage the gel, which is inside the condom teat, onto the head of the penis for 15 seconds.**<br>• Cautions about tearing accidentally with fingernails or rings.<br>• If the condom starts to roll back up during sex, it should be rolled back down straight away. If the man feels the condom slip or tighten excessively, he should stop and check the condom placement to avoid it	☐

breaking. If the condom comes off the penis, the man should stop and put on a standard (not a Futura Max) condom.

6. Counsels about what to do if condom breaks or slips off during sex. ☐
    - Woman should return to clinic to be assessed for emergency contraception.
    - Woman should request emergency contraceptive pills within 72 hours (the earlier the better) of unprotected sex or breakage of a condom.

7. Has patient demonstrate and practice putting on condom using the model, banana or fingers. ☐
    - Corrects any technique errors.

8. Counsels patient on how to remove penis from vagina with condom intact and without spilling semen. ☐
    - Hold on to rim of condom when withdrawing.
    - Be careful not to let semen spill into vagina when penis is flaccid.

9. Discusses use of lubricants and what not to use. ☐
    - Do not use any of these: petroleum-based products (e.g., Vaseline); mineral, vegetable, or cooking oil; baby oil; margarine or butter.

10. Advises to use a spermicide or water-based lubricant if one is needed. ☐

11. Advises patient to dispose of condoms by burning, burying, or throwing in the latrine. ☐

12. Repeats main condom messages to patient. ☐
    - Be sure to have condom on hand before you need it.
    - Use a condom with every act of intercourse.
    - Do not use a single condom more than once.
    - Do not rely on condom if package is damaged, torn, outdated, dry, brittle or sticky.
    - **Futura Max condoms are designed to make sex more fun for the couple. Futura Max condoms contain a special gel that makes the man's erection get harder and last longer. This can make sex more pleasurable for the couple.**

13. Provides patient with condoms for two months. ☐
    - Multiply patient's expected weekly coital frequency by 9. Give this number of condoms or, if this number is less than 20, give 20 condoms.
    - Give 2 standard condoms to use in case the couple has sex more than once within 24 hours.

14. Instructs patient not to share study condoms with anyone but main partner. ☐
    - Do not share study condoms with family or friends.

15. Reassures patient that should return at any time for advice or more condoms. ☐

VN CSD500 Study
Futura Max™ Information Sheet

Futura Max™ condoms are natural rubber latex condoms which contain Zanifil® gel. They have been shown to maximize hardness by stimulating blood flow within the penis. This can help prevent loss or partial loss of erection, ensuring an intense experience for both you and your partner.

How to use: Use a condom only once. Condoms used more than once are more likely to slip or break and not provide adequate protection. Infections can be passed on in bodily fluids present in the condom after use.

Image redacted due to copyright

1. Tear open the foil and remove the condom. Be careful with sharp objects such as nails and jewelry. If the carton or foil is damaged, discard and use a condom from an undamaged package.

Image redacted due to copyright

2. Put the condom on the erect penis before any contact occurs between the penis and the partner's body. This will help prevent sexually transmitted infections and pregnancy. Squeezing the teat at the end of the condom, ensuring no air is trapped and that the Zanifil® gel is on the inside, place the condom on the top of the penis and fully roll it down the shaft with the other hand.

Image redacted due to copyright

3. **Once the condom is on the penis, squeeze the teat of the condom and massage the gel, which is inside the condom teat, onto the head of the penis for 15 seconds. Ensure not to damage the condom by sharp objects such as jewelry or fingernails.** If the condom starts to roll back up during sex, roll it back down straight away. If you feel the condom slipping or tightening excessively stop and check – or it may break. If it comes off, stop and put on a standard condom.

Image redacted due to copyright

4. Withdraw soon after ejaculation, while the penis is still erect, holding the condom in place at the rim.

5. Dispose of the used condom in a bin. Never flush it down the toilet.

Important warnings:
The active ingredient, Glyceryl Trinitrate 1%, can cause headaches, faintness and nausea, loss of sensation, dizziness and skin irritation. If you experience any of these symptoms, stop using **Futura Max™** condoms. If your symptoms continue, contact your doctor.

Don't use Futura Max™ condoms if:

- You or your partner are taking medication to lower your blood pressure or for male impotence, migraines, headaches, glaucoma or anemia.
- The condom is likely to come into contact with inflamed or broken skin.
- You are pregnant or breast feeding.

Consult your doctor before use if you or your partner:

- Have low blood pressure or a pre-existing heart condition;
- Are using medicines that could come into contact with the condom;
- Might be sensitive or allergic to latex.

Important information for Futura Max™ condoms:

- Don't use more than one condom in a 24 hour period.
- Don't use for oral sex.
- For anal sex, use additional lubricant on the outside of the condom to help reduce the risk of breaking or slipping.
- No method of contraception can provide 100% effectiveness against pregnancy or the transmission of HIV or STIs.
- If you are concerned that your partner may have become pregnant or infected, contact your doctor immediately.
- Seek medical advice as soon as possible, at least within 72 hours, if a condom you are using breaks or leaks.
- Use only additional lubricants that are compatible with latex condoms.
- Don't use with oil-based lubricants such as petroleum jelly, baby oil, body lotions, massage oils, butter or margarin
