# Supplementary material for: Effectiveness of erectogenic condom against semen exposure among women in Vietnam: Randomized controlled trial
Source: PLoS One. 2022 Feb 17;17(2):e0263503. doi: 10.1371/journal.pone.0263503 (PMC8853499; doi:10.1371/journal.pone.0263503)
Supplement: S2 File — (DOC) [file pone.0263503.s003.doc]

**VN CSD500 Study**

**Randomization Assignment Form**

| **DCF #**  **VNCSD 02** | **Staff code**  **|____||____||____||____|** | **Visit date**  **|____||____|/|____||____|/ 201|____|**  **Day Month Year** | **PIN**  **|____||____||____||____|** |
| --- | --- | --- | --- |

| **No.** | **Question** | **Responses** |  |
| --- | --- | --- | --- |
|  | Intervention arm assignment: | [___] Control arm 0  [___] Intervention arm 1 |  |

**VN CSD500 Study**

**Vaginal Swab Collection Form**

| **DCF #**  **VNCSD 03** | **Staff code**  **|____||____||____||____|** | **Visit date**  **|____||____|/|____||____|/ 201|____|**  **Day Month Year** | **PIN**  **|____||____||____||____|** |
| --- | --- | --- | --- |

| **No.** | **Question** | **Responses** | **Skip to** |
| --- | --- | --- | --- |
|  | Study visit: | [___] Enrollment 1  [___] 2-Month 2  [___] 4-Month 3  [___] 6-Month 4 |  |
|  | Was the vaginal swab collected? | [___] No, specify reason 0  ____________________________________  [___] Yes 1 |  |

**VN CSD500 Study**

**Female Enrollment Questionnaire**

| **DCF #**  **VNCSD 04** | **Staff code**  **|____||____||____||____|** | **Visit date**  **|____||____|/|____||____|/ 201|____|**  **Day Month Year** | **PIN**  **|____||____||____||____|** |
| --- | --- | --- | --- |

Now, I’m going to ask you questions for a survey. Please answer as honestly as you can. Your responses will be kept confidential and will not be shown to other persons.

| **No.** | **Question** | | **Responses** | | | | | | | | **Skip to** |
| --- | --- | --- | --- | --- | --- | --- | --- | --- | --- | --- | --- |
|  | Do you live in a… | | [___] City (50,000 or greater population) 1  [___] Town 2  [___] Countryside / rural area 3  [___] Decline 99 | | | | | | | |  |
|  | What is the main source of drinking water for members of your household? | | [___] Piped water into residence/plot 1  [___] Piped water to public tap 2  [___] Well into residence/plot 3  [___] Public well 4  [___] Surface water (spring, river, stream, lake, pond, dam) 5  [___] Rain water 6  [___] Tanker truck 7  [___] Bottled water 8  [___] Other, specify 9  ____________________________________  [___] Decline 99 | | | | | | | |  |
|  | What kind of toilet facility do members of your household usually use? | | [___] Flush toilet 1  [___] Pit latrine ventilated 2  [___] Improved pit toilet/latrine 3  [___] Traditional pit toilet/latrine 4  [___] No facility/bush/field 5  [___] Other, specify 6  ____________________________________  [___] Decline 99 | | | | | | | |  |
|  | Do you share this toilet facility with other households? | | [___] No 0  [___] Yes 1  [___] Decline 99 | | | | | | | |  |
|  | Does your household have any of these:  ***Read entire list.***  ***Mark all that apply.*** | | [___] Electricity 1  [___] A television 2  [___] A telephone (any kind) 3  [___] A refrigerator 4  [___] A washing machine 5  [___] A water pump 6  [___] A cupboard 7  [___] A table and chairs 8  [___] A bicycle 9  [___] A motorcycle or motor scooter 10  [___] An animal-drawn cart 11  [___] A car or truck 12  [___] A boat with a motor 13  [___] A boat without a motor 14  [___] None of the above 15  [___] Decline 99 | | | | | | | |  |
|  | What type of fuel does your household mainly use for cooking?  ***Read list if needed.*** | | [___] Electricity 1  [___] Gas 2  [___] Ranger – gas and electric 3  [___] Kerosene 4  [___] Coal 5  [___] Wood 6  [___] Straw 7  [___] Other, specify 8  ____________________________________  [___] Decline 99 | | | | | | | |  |
|  | How many rooms in your household are used for sleeping? | | |__|__| Rooms  [___] Decline 99 | | | | | | | |  |
|  | Have you ever attended school? | | [___] No 0  [___] Yes 1  [___] Decline 99 | | | | | | | | **🡪12** |
|  | Are you currently a student? | | [___] No 0  [___] Yes 1  [___] Decline 99 | | | | | | | |  |
|  | What was the highest level of school you have attended? | | [___] Primary 1  [___] Lower secondary 2  [___] Upper secondary 3  [___] Higher 4  [___] Decline 99 | | | | | | | |  |
|  | Aside from your own housework, do you currently work? | | [___] No 0  [___] Yes 1  [___] Decline 99 | | | | | | | | **🡪13** |
|  | As you know, some women take up jobs for which they are paid in cash or kind. Other sell things, have a small business or work on the family farm or in the family business. Do you currently do these or any other work? | | [___] No 0  [___] Yes 1  [___] Decline 99 | | | | | | | | **🡪14** |
|  | What kind of work do you mainly do? | | [___] Unskilled laborer 1  [___] Semi-skilled / clerical 2  [___] Skilled laborer 3  [___] Professional 4  [___] Other, specify 5  ____________________________________  [___] Decline 99 | | | | | | | |  |
|  | What is your religion? | | [___] No religion 1  [___] Buddhist 2  [___] Catholic 3  [___] Protestant 4  [___] Cao Dai 5  [___] Hoa Hao 6  [___] Islam 7  [___] Other, specify 8  ____________________________________  [___] Decline 99 | | | | | | | |  |
|  | What ethnic group do you belong to? | | [___] Kinh 1  [___] Tay 2  [___] Thai 3  [___] Chinese 4  [___] Khmer 5  [___] Muong 6  [___] Nung 7  [___] Hre 8  [___] Phu la 9  [___] Ede 10  [___] Dao 11  [___] Cotu 12  [___] Cham 13  [___] Other, specify 14  ____________________________________  [___] Decline 99 | | | | | | | |  |
| Now I am going to ask you some questions about pregnancy and sexual activity in order to gain a better understanding of some family life issues. | | | | | | | | | | | |
|  | Have you ever used any of these other methods to prevent pregnancy?  ***Read entire list.***  ***Mark all that apply.*** | | [___] Pill 1  [___] IUD 2  [___] Injections 3  [___] Implants 4  [___] Diaphragm, foam, jelly 5  [___] Male partner sterilization 6  [___] Rhythm, period abstinence 7  [___] Withdrawal 8  [___] Other, specify 9  ____________________________________  [___] Decline 99 | | | | | | | |  |
|  | How important is it to you to not become pregnant in the next 6 months?  ***Read response options.*** | | [___] Very important 1  [___] Important 2  [___] Neutral / no opinion 3  [___] Decline 99 | | | | | | | |  |
|  | Have you ever been pregnant? | | [___] No 0  [___] Yes 1  [___] Decline 99 | | | | | | | | **🡪21** |
|  | How many times and how did they end?  ***Read entire list.***  ***Mark response for each line.***  ***Total should equal sum of other responses.***  ***Probe to ensure all recalled.*** | | |__|__| Live births  |__|__| Still births  |__|__| Induced abortions / menstrual regulation  |__|__| Spontaneous abortions  |__|__| Total  [___] Decline 99 | | | | | | | |  |
|  | How recent was your last pregnancy? | | [___] Within the past month 0  [___] Within the past 6 months but not in the past month 1  [___] Within the past 12 months but not in the past 6 months 2  [___] More than 12 months ago 3 | | | | | | | |  |
|  | How old were you when you have sexual intercourse for the very first time? | | |__|__| Years  [___] Decline 99 | | | | | | | |  |
|  | How old was the person you first had sexual intercourse with? | | |__|__| Years  [___] Don’t know 98  [___] Decline 99 | | | | | | | | **🡪25** |
|  | Was this person older than you, younger than you, or about the same age as you? | | [___] Older 1  [___] Younger 2  [___] About the same age 3  [___] Don’t know 98  [___] Decline 99 | | | | | | | | **🡪25**  **🡪25**  **🡪25** |
|  | Would you say this person was ten or more years older than you or less than ten years older than you? | | [___] Ten or more years older 1  [___] Less than ten years older 2  [___] Don’t know 98  [___] Decline 99 | | | | | | | |  |
|  | How many male sex partners have you had in your lifetime?  Count every guy, even those you had sex with only once or twice. | | |__|__| Men  [___] Decline 99 | | | | | | | |  |
|  | In the past year, did you have more than one sexual relationship at the same time?  ***Probe for overlapping (concurrent) partnerships.*** | | [___] No 0  [___] Yes 1  [___] Decline 99 | | | | | | | |  |
|  | Were you forced or pressured by anyone to have sex even though you did not want to have sex? | | [___] No 0  [___] Yes 1  [___] Decline 99 | | | | | | | |  |
|  | Now think about your current partner ***<name>.***  Is ***<name>*** living with you now or is he staying elsewhere? | | [___] Living together 1  [___] Staying elsewhere 2  [___] Decline 99 | | | | | | | |  |
|  | When was the last time you had vaginal sex with him?  By vaginal sex, I mean sex in which the penis goes into the vagina. | | [___] Today 1  [___] Yesterday 2  [___] Day before yesterday 3  [___] More than two days ago but less than one week 4  [___] One week or more but less than two weeks 5  [___] More than two weeks ago 6  [___] Decline 99 | | | | | | | |  |
|  | Did you use a condom for this last act with ***<name>***? | | [___] No 0  [___] Yes 1  [___] Decline 99 | | | | | | | | **🡪32** |
|  | When was the last time you had vaginal sex with him without using a condom for the entire act? | | [___] Today 1  [___] Yesterday 2  [___] Day before yesterday 3  [___] More than two days ago but less than one week 4  [___] One week or more but less than two weeks 5  [___] More than two weeks ago 6  [___] Never had sex with him without a condom 7  [___] Decline 99 | | | | | | | | **🡪33** |
|  | During the past month, how often did you use a condom when you had vaginal sex with ***<name>***? | | [___] Always 1  [___] More than half of the time 2  [___] About half of the time 3  [___] Less than half of the time 4  [___] Never 5  [___] Decline 99 | | | | | | | |  |
|  | How likely are you to get a sexually transmitted disease from ***<name>*** if you have sex with him without using a condom? | | [___] Very likely 1  [___] Likely 2  [___] Unlikely 3  [___] Very unlikely 4  [___] Decline 99 | | | | | | | |  |
| Now think about the last time you had sex with ***<name>***. Would you say that it… | | | | | | | | | | | |
|  | Was very bad | 1 | | 2 | 3 | 4 | 5 | 6 | 7 | Was very good | |
|  | Was not at all what I wanted it to be | 1 | | 2 | 3 | 4 | 5 | 6 | 7 | Was very much what I wanted it to be | |
|  | Didn’t meet my expectations at all | 1 | | 2 | 3 | 4 | 5 | 6 | 7 | Exceeded my expectation | |
|  | Was not at all pleasurable | 1 | | 2 | 3 | 4 | 5 | 6 | 7 | Was very pleasurable | |
|  | Was sex that I was not into at all | 1 | | 2 | 3 | 4 | 5 | 6 | 7 | Was sex that I was totally into | |
|  | Very bad physically | 1 | | 2 | 3 | 4 | 5 | 6 | 7 | Very good physically | |
|  | Very bad emotionally | 1 | | 2 | 3 | 4 | 5 | 6 | 7 | Very good emotionally | |
|  | Was the sex act…  ***Read response options.*** | | [___] Too short 1  [___] Neither too short nor too long 2  [___] Too long 3 | | | | | | | |  |
|  | When was the last time you had  4 or more drinks containing alcohol in one day? | | [___] Less than one week ago 1  [___] One week ago or more but less than one month ago 2  [___] One month ago or more but less than one year ago 3  [___] Never 4  [___] Decline 99 | | | | | | | |  |
|  | During the past 30 days, did you use any drugs such as marijuana, ecstasy, crack, cocaine, speed, meth, or heroin? | | [___] No 1  [___] Yes 2  [___] Decline 99 | | | | | | | |  |
|  | Has a doctor or other medical provider ever told you that you had a sexually transmitted disease? | | [___] No 1  [___] Yes 2  [___] Decline 99 | | | | | | | |  |
| These next questions ask about your relationship with ***<name>***? | | | | | | | | | | | |
|  | If I asked ***<name>*** to use a condom, he would get violent.  ***Read response options.*** | | [___] Strongly agree 1  [___] Agree 2  [___] Disagree 3  [___] Strongly disagree 4  [___] Decline 99 | | | | | | | |  |
|  | If I asked him to use a condom, he would get angry.  ***Read response options.*** | | [___] Strongly agree 1  [___] Agree 2  [___] Disagree 3  [___] Strongly disagree 4  [___] Decline 99 | | | | | | | |  |
|  | Most of the time, we do what he wants to do.  ***Read response options.*** | | [___] Strongly agree 1  [___] Agree 2  [___] Disagree 3  [___] Strongly disagree 4  [___] Decline 99 | | | | | | | |  |
|  | He won’t let me wear certain things.  ***Read response options.*** | | [___] Strongly agree 1  [___] Agree 2  [___] Disagree 3  [___] Strongly disagree 4  [___] Decline 99 | | | | | | | |  |
|  | When ***<name>*** and I are together, I’m quieter.  ***Read response options.*** | | [___] Strongly agree 1  [___] Agree 2  [___] Disagree 3  [___] Strongly disagree 4  [___] Decline 99 | | | | | | | |  |
|  | He has more say than I do about important decisions that affect us.  ***Read response options.*** | | [___] Strongly agree 1  [___] Agree 2  [___] Disagree 3  [___] Strongly disagree 4  [___] Decline 99 | | | | | | | |  |
|  | He tells me who I can spend time with.  ***Read response options.*** | | [___] Strongly agree 1  [___] Agree 2  [___] Disagree 3  [___] Strongly disagree 4  [___] Decline 99 | | | | | | | |  |
|  | If I asked him to use a condom, he would think I’m having sex with other people.  ***Read response options.*** | | [___] Strongly agree 1  [___] Agree 2  [___] Disagree 3  [___] Strongly disagree 4  [___] Decline 99 | | | | | | | |  |
|  | I feel stuck in our relationship.  ***Read response options.*** | | [___] Strongly agree 1  [___] Agree 2  [___] Disagree 3  [___] Strongly disagree 4  [___] Decline 99 | | | | | | | |  |
|  | He does what he wants, even if I do not want him to.  ***Read response options.*** | | [___] Strongly agree 1  [___] Agree 2  [___] Disagree 3  [___] Strongly disagree 4  [___] Decline 99 | | | | | | | |  |
|  | I am more committed to our relationship than he is.  ***Read response options.*** | | [___] Strongly agree 1  [___] Agree 2  [___] Disagree 3  [___] Strongly disagree 4  [___] Decline 99 | | | | | | | |  |
|  | When ***<name>*** and I disagree, he gets his way most of the time.  ***Read response options.*** | | [___] Strongly agree 1  [___] Agree 2  [___] Disagree 3  [___] Strongly disagree 4  [___] Decline 99 | | | | | | | |  |
|  | He gets more out of our relationship than I do.  ***Read response options.*** | | [___] Strongly agree 1  [___] Agree 2  [___] Disagree 3  [___] Strongly disagree 4  [___] Decline 99 | | | | | | | |  |
|  | He always wants to know where I am.  ***Read response options.*** | | [___] Strongly agree 1  [___] Agree 2  [___] Disagree 3  [___] Strongly disagree 4  [___] Decline 99 | | | | | | | |  |
|  | He might be having sex with someone else.  ***Read response options.*** | | [___] Strongly agree 1  [___] Agree 2  [___] Disagree 3  [___] Strongly disagree 4  [___] Decline 99 | | | | | | | |  |
|  | Who usually has more say about whose friends to go out with?  ***Read response options.*** | | [___] Him 1  [___] Both of you equally 2  [___] You 3  [___] Decline 99 | | | | | | | |  |
|  | Who usually has more say about whether you have sex?  ***Read response options.*** | | [___] Him 1  [___] Both of you equally 2  [___] You 3  [___] Decline 99 | | | | | | | |  |
|  | Who usually has more say about what you do together?  ***Read response options.*** | | [___] Him 1  [___] Both of you equally 2  [___] You 3  [___] Decline 99 | | | | | | | |  |
|  | Who usually has more say about how often you see one another?  ***Read response options.*** | | [___] Him 1  [___] Both of you equally 2  [___] You 3  [___] Decline 99 | | | | | | | |  |
|  | Who usually has more say about when you talk about serious things?  ***Read response options.*** | | [___] Him 1  [___] Both of you equally 2  [___] You 3  [___] Decline 99 | | | | | | | |  |
|  | In general, who do you think has more power in your relationship?  ***Read response options.*** | | [___] Him 1  [___] Both of you equally 2  [___] You 3  [___] Decline 99 | | | | | | | |  |
|  | Who usually has more say about whether you use condoms?  ***Read response options.*** | | [___] Him 1  [___] Both of you equally 2  [___] You 3  [___] Decline 99 | | | | | | | |  |
|  | Who usually has more say about what types of sexual acts you do?  ***Read response options.*** | | [___] Him 1  [___] Both of you equally 2  [___] You 3  [___] Decline 99 | | | | | | | |  |
|  | Who usually has more say about how you spend your household’s money?  ***Read response options.*** | | [___] Him 1  [___] Both of you equally 2  [___] You 3  [___] Decline 99 | | | | | | | |  |
|  | ***Do not ask if Q30 = Yes or if Q32 = 1,2,3, or 4***  Have you ever had a partner use a condom during sex? | | [___] No 1  [___] Yes 2  [___] Decline 99 | | | | | | | |  |
| Finally, I’m going to read a set of statements and for each one please tell me whether you: strongly disagree, mostly disagree, somewhat disagree, neutral/no opinion, somewhat agree, mostly agree, or strongly agree. | | | | | | | | | | | |

|  | I do not hesitate to embrace new experiences if I think they can improve my health.  ***Read response options.*** | [___] Strongly disagree 1  [___] Mostly disagree 2  [___] Somewhat disagree 3  [___] Neutral / no opinion 4  [___] Somewhat agree 5  [___] Mostly agree 6  [___] Strongly agree 7  [___] Decline 99 |  |
| --- | --- | --- | --- |
|  | I frequently think about the health problems I may have in the future.  ***Read response options.*** | [___] Strongly disagree 1  [___] Mostly disagree 2  [___] Somewhat disagree 3  [___] Neutral / no opinion 4  [___] Somewhat agree 5  [___] Mostly agree 6  [___] Strongly agree 7  [___] Decline 99 |  |
|  | When I implement a health behavior, it's because I want to protect myself from getting sick.  ***Read response options.*** | [___] Strongly disagree 1  [___] Mostly disagree 2  [___] Somewhat disagree 3  [___] Neutral / no opinion 4  [___] Somewhat agree 5  [___] Mostly agree 6  [___] Strongly agree 7  [___] Decline 99 |  |
|  | If I succeed in reaching a health goal, this motivates me to go further.  ***Read response options.*** | [___] Strongly disagree 1  [___] Mostly disagree 2  [___] Somewhat disagree 3  [___] Neutral / no opinion 4  [___] Somewhat agree 5  [___] Mostly agree 6  [___] Strongly agree 7  [___] Decline 99 |  |
|  | I think that taking care of my health is pleasurable.  ***Read response options.*** | [___] Strongly disagree 1  [___] Mostly disagree 2  [___] Somewhat disagree 3  [___] Neutral / no opinion 4  [___] Somewhat agree 5  [___] Mostly agree 6  [___] Strongly agree 7  [___] Decline 99 |  |
|  | I see myself as someone who does my utmost to improve my health.  ***Read response options.*** | [___] Strongly disagree 1  [___] Mostly disagree 2  [___] Somewhat disagree 3  [___] Neutral / no opinion 4  [___] Somewhat agree 5  [___] Mostly agree 6  [___] Strongly agree 7  [___] Decline 99 |  |
|  | I often worry about mistakes I could make concerning my health.  ***Read response options.*** | [___] Strongly disagree 1  [___] Mostly disagree 2  [___] Somewhat disagree 3  [___] Neutral / no opinion 4  [___] Somewhat agree 5  [___] Mostly agree 6  [___] Strongly agree 7  [___] Decline 99 |  |
|  | If I see a good opportunity to improve my health, I take advantage of it right away.  ***Read response options.*** | [___] Strongly disagree 1  [___] Mostly disagree 2  [___] Somewhat disagree 3  [___] Neutral / no opinion 4  [___] Somewhat agree 5  [___] Mostly agree 6  [___] Strongly agree 7  [___] Decline 99 |  |
| Thank you very much for answering these questions. Do you have any questions for me? | | | |

**VN CSD500 Study**

**Male Enrollment Questionnaire**

| **DCF #**  **VNCSD 05** | **Staff code**  **|____||____||____||____|** | **Visit date**  **|____||____|/|____||____|/ 201|____|**  **Day Month Year** | **PIN**  **|____||____||____||____|** |
| --- | --- | --- | --- |

Now, I’m going to ask you questions for a survey. Please answer as honestly as you can. Your responses will be kept confidential and will not be shown to other persons.

| **No.** | **Question** | | **Responses** | | | | | | | | **Skip to** |
| --- | --- | --- | --- | --- | --- | --- | --- | --- | --- | --- | --- |
|  | Have you ever attended school? | | [___] No 0  [___] Yes 1  [___] Decline 99 | | | | | | | | **🡪5** |
|  | Are you currently a student? | | [___] No 0  [___] Yes 1  [___] Decline 99 | | | | | | | |  |
|  | What was the highest level of school you have attended? | | [___] Primary 1  [___] Lower secondary 2  [___] Upper secondary 3  [___] Higher 4  [___] Decline 99 | | | | | | | |  |
|  | Do you currently work? | | [___] No 1  [___] Yes, full time 2  [___] Yes, part- time 3  [___] Decline 99 | | | | | | | | **🡪6**  **🡪6** |
|  | What have you been doing for most of the time over the last 12 months? | | [___] Going to school/studying 1  [___] Looking for work 2  [___] Retired 3  [___] Too ill to work 4  [___] Handicapped, cannot work 5  [___] Housework/child care 6  [___] Other, specify 7  ____________________________________  [___] Decline 99 | | | | | | | | **🡪7**  **🡪7**  **🡪7**  **🡪7**  **🡪7**  **🡪7**  **🡪7** |
|  | What kind of work do you mainly do? | | [___] Unskilled laborer 1  [___] Semi-skilled / clerical 2  [___] Skilled laborer 3  [___] Professional 4  [___] Other, specify 5  ____________________________________  [___] Decline 99 | | | | | | | |  |
|  | What is your religion? | | [___] No religion 1  [___] Buddhist 2  [___] Catholic 3  [___] Protestant 4  [___] Cao Dai 5  [___] Hoa Hao 6  [___] Islam 7  [___] Other, specify 8  ____________________________________  [___] Decline 99 | | | | | | | |  |
|  | What ethnic group do you belong to? | | [___] Kinh 1  [___] Tay 2  [___] Thai 3  [___] Chinese 4  [___] Khmer 5  [___] Muong 6  [___] Nung 7  [___] Hre 8  [___] Phu la 9  [___] Ede 10  [___] Dao 11  [___] Cotu 12  [___] Cham 13  [___] Other, specify 14  ____________________________________  [___] Decline 99 | | | | | | | |  |
| Now I am going to ask you some questions about pregnancy and sexual activity in order to gain a better understanding of some family life issues. | | | | | | | | | | | |
|  | How important is it to you that your partner ***<name>*** not become pregnant in the next 6 months?  ***Read response options.*** | | [___] Very important 1  [___] Important 2  [___] Neutral / no opinion 3  [___] Decline 99 | | | | | | | |  |
|  | How old were you when you have sexual intercourse for the very first time? | | |__|__| Years  [___] Decline 99 | | | | | | | |  |
|  | How many female sex partners have you had in your lifetime?  Count every woman, even those you had sex with only once or twice. | | |__|__| Women  [___] Decline 99 | | | | | | | |  |
|  | How many male sex partners have you had in your lifetime?  Count every man, even those you had sex with only once or twice. | | |__|__| Men  [___] Decline 99 | | | | | | | |  |
|  | In the past year, did you have more than one sexual relationship at the same time?  ***Probe for overlapping (concurrent) partnerships.*** | | [___] No 0  [___] Yes 1  [___] Decline 99 | | | | | | | |  |
|  | Did you ever pay someone money or gifts for sex? | | [___] No 0  [___] Yes 1  [___] Decline 99 | | | | | | | |  |
|  | When was the last time you had vaginal sex with ***<name>***?  By vaginal sex, I mean sex in which the penis goes into the vagina. | | [___] Today 1  [___] Yesterday 2  [___] Day before yesterday 3  [___] More than two days ago but less than one week 4  [___] One week or more but less than two weeks 5  [___] More than two weeks ago 6  [___] Decline 99 | | | | | | | |  |
|  | Did you use a condom for this last act with ***<name>***? | | [___] No 0  [___] Yes 1  [___] Decline 99 | | | | | | | | **🡪18** |
|  | When was the last time you had vaginal sex with her without using a condom for the entire act? | | [___] Today 1  [___] Yesterday 2  [___] Day before yesterday 3  [___] More than two days ago but less than one week 4  [___] One week or more but less than two weeks 5  [___] More than two weeks ago 6  [___] Never had sex with partner without using a condom 7  [___] Decline 99 | | | | | | | | **🡪19** |
|  | During the past month, how often did you use a condom when you had vaginal sex with ***<name>***? | | [___] Always 1  [___] More than half of the time 2  [___] About half of the time 3  [___] Less than half of the time 4  [___] Never 5  [___] Decline 99 | | | | | | | | **🡪20** |
|  | In the past month when you used a condom with ***<name>***, was the condom on for the whole sex act, from beginning to end? | | [___] Never 1  [___] Sometimes 2  [___] Always 3  [___] Decline 99 | | | | | | | |  |
|  | ***Do not ask if Q16 = Yes or if Q18 = 1,2,3, or 4***  Have you ever used a condom with any partner? | | [___] No 0  [___] Yes 1  [___] Decline 99 | | | | | | | |  |
|  | Have you ever used withdrawal? By withdrawal, I mean that no condom is used and the penis is pulled out before ejaculation. | | [___] No 0  [___] Yes 1  [___] Don’t know 88  [___] Decline 99 | | | | | | | | **🡪24** |
|  | In the past month, how often did you and ***<name>*** use withdrawal? | | [___] Never 1  [___] Sometimes 2  [___] Always 3  [___] Don’t know 88  [___] Decline 99 | | | | | | | |  |
|  | How likely are you to get a sexually transmitted disease from ***<name>*** if you have sex with her without using a condom? | | [___] Very likely 1  [___] Likely 2  [___] Unlikely 3  [___] Very unlikely 4  [___] Decline 99 | | | | | | | |  |
| Now, I’m going to read some statements about condoms. Please tell me if you strongly agree, agree, neither agree nor disagree, disagree or strongly disagree with the statement. | | | | | | | | | | | |
|  | Most of the time neither of us has a condom available.  ***Read response options if needed.*** | | [___] Strongly agree 1  [___] Agree 2  [___] Neither agree nor disagree 3  [___] Disagree 4  [___] Strongly disagree 5 | | | | | | | |  |
|  | I don’t want my partner to put a condom on me.  ***Read response options if needed.*** | | [___] Strongly agree 1  [___] Agree 2  [___] Neither agree nor disagree 3  [___] Disagree 4  [___] Strongly disagree 5 | | | | | | | |  |
|  | If I suggested to my partner we use a condom, she might end the relationship.  ***Read response options if needed.*** | | [___] Strongly agree 1  [___] Agree 2  [___] Neither agree nor disagree 3  [___] Disagree 4  [___] Strongly disagree 5 | | | | | | | |  |
|  | I can never find a condom right before sexual intercourse.  ***Read response options if needed.*** | | [___] Strongly agree 1  [___] Agree 2  [___] Neither agree nor disagree 3  [___] Disagree 4  [___] Strongly disagree 5 | | | | | | | |  |
|  | My partner doesn’t want us to use condoms.  ***Read response options if needed.*** | | [___] Strongly agree 1  [___] Agree 2  [___] Neither agree nor disagree 3  [___] Disagree 4  [___] Strongly disagree 5 | | | | | | | |  |
|  | If I suggested we use a condom, my partner would think I don’t trust her.  ***Read response options if needed.*** | | [___] Strongly agree 1  [___] Agree 2  [___] Neither agree nor disagree 3  [___] Disagree 4  [___] Strongly disagree 5 | | | | | | | |  |
|  | I usually forget about using condoms.  ***Read response options if needed.*** | | [___] Strongly agree 1  [___] Agree 2  [___] Neither agree nor disagree 3  [___] Disagree 4  [___] Strongly disagree 5 | | | | | | | |  |
|  | If I suggested we use a condom, my partner would think I’m accusing her of cheating.  ***Read response options if needed.*** | | [___] Strongly agree 1  [___] Agree 2  [___] Neither agree nor disagree 3  [___] Disagree 4  [___] Strongly disagree 5 | | | | | | | |  |
|  | I would be afraid to suggest to my partner we use a condom.  ***Read response options if needed.*** | | [___] Strongly agree 1  [___] Agree 2  [___] Neither agree nor disagree 3  [___] Disagree 4  [___] Strongly disagree 5 | | | | | | | |  |
|  | If I used a condom, my partner might think I’m cheating on her.  ***Read response options if needed.*** | | [___] Strongly agree 1  [___] Agree 2  [___] Neither agree nor disagree 3  [___] Disagree 4  [___] Strongly disagree 5 | | | | | | | |  |
|  | Condoms rub and cause irritation.  ***Read response options if needed.*** | | [___] Strongly agree 1  [___] Agree 2  [___] Neither agree nor disagree 3  [___] Disagree 4  [___] Strongly disagree 5 | | | | | | | |  |
|  | I wouldn’t know where to get a condom.  ***Read response options if needed.*** | | [___] Strongly agree 1  [___] Agree 2  [___] Neither agree nor disagree 3  [___] Disagree 4  [___] Strongly disagree 5 | | | | | | | |  |
|  | Condoms don’t feel good.  ***Read response options if needed.*** | | [___] Strongly agree 1  [___] Agree 2  [___] Neither agree nor disagree 3  [___] Disagree 4  [___] Strongly disagree 5 | | | | | | | |  |
|  | If I suggested my partner use a condom, she might be turned off and lose interest in having sex.  ***Read response options if needed.*** | | [___] Strongly agree 1  [___] Agree 2  [___] Neither agree nor disagree 3  [___] Disagree 4  [___] Strongly disagree 5 | | | | | | | |  |
|  | Condoms interrupt the mood.  ***Read response options if needed.*** | | [___] Strongly agree 1  [___] Agree 2  [___] Neither agree nor disagree 3  [___] Disagree 4  [___] Strongly disagree 5 | | | | | | | |  |
|  | Condoms are against my religious values.  ***Read response options if needed.*** | | [___] Strongly agree 1  [___] Agree 2  [___] Neither agree nor disagree 3  [___] Disagree 4  [___] Strongly disagree 5 | | | | | | | |  |
|  | Condoms feel unnatural.  ***Read response options if needed.*** | | [___] Strongly agree 1  [___] Agree 2  [___] Neither agree nor disagree 3  [___] Disagree 4  [___] Strongly disagree 5 | | | | | | | |  |
|  | If I used a condom, my partner might get angry.  ***Read response options if needed.*** | | [___] Strongly agree 1  [___] Agree 2  [___] Neither agree nor disagree 3  [___] Disagree 4  [___] Strongly disagree 5 | | | | | | | |  |
|  | Condoms don’t fit right.  ***Read response options if needed.*** | | [___] Strongly agree 1  [___] Agree 2  [___] Neither agree nor disagree 3  [___] Disagree 4  [___] Strongly disagree 5 | | | | | | | |  |
|  | Condoms cost too much.  ***Read response options if needed.*** | | [___] Strongly agree 1  [___] Agree 2  [___] Neither agree nor disagree 3  [___] Disagree 4  [___] Strongly disagree 5 | | | | | | | |  |
|  | I don’t have transportation to buy or get condoms.  ***Read response options if needed.*** | | [___] Strongly agree 1  [___] Agree 2  [___] Neither agree nor disagree 3  [___] Disagree 4  [___] Strongly disagree 5 | | | | | | | |  |
|  | I would be embarrassed to buy condoms or ask for them.  ***Read response options if needed.*** | | [___] Strongly agree 1  [___] Agree 2  [___] Neither agree nor disagree 3  [___] Disagree 4  [___] Strongly disagree 5 | | | | | | | |  |
|  | It’s up to the man to provide a condom.  ***Read response options if needed.*** | | [___] Strongly agree 1  [___] Agree 2  [___] Neither agree nor disagree 3  [___] Disagree 4  [___] Strongly disagree 5 | | | | | | | |  |
|  | I feel closer to my partner without a condom.  ***Read response options if needed.*** | | [___] Strongly agree 1  [___] Agree 2  [___] Neither agree nor disagree 3  [___] Disagree 4  [___] Strongly disagree 5 | | | | | | | |  |
|  | If I suggested we use a condom, she might think I’m putting her down or insulting her.  ***Read response options if needed.*** | | [___] Strongly agree 1  [___] Agree 2  [___] Neither agree nor disagree 3  [___] Disagree 4  [___] Strongly disagree 5 | | | | | | | |  |
|  | I don’t need to use a condom, I never catch anything.  ***Read response options if needed.*** | | [___] Strongly agree 1  [___] Agree 2  [___] Neither agree nor disagree 3  [___] Disagree 4  [___] Strongly disagree 5 | | | | | | | |  |
|  | When I use a condom I feel less involved or committed to the relationship.  ***Read response options if needed.*** | | [___] Strongly agree 1  [___] Agree 2  [___] Neither agree nor disagree 3  [___] Disagree 4  [___] Strongly disagree 5 | | | | | | | |  |
|  | Condoms change the climax or orgasm.  ***Read response options if needed.*** | | [___] Strongly agree 1  [___] Agree 2  [___] Neither agree nor disagree 3  [___] Disagree 4  [___] Strongly disagree 5 | | | | | | | |  |
|  | I don’t need to use a condom, I use another method.  ***Read response options if needed.*** | | [___] Strongly agree 1  [___] Agree 2  [___] Neither agree nor disagree 3  [___] Disagree 4  [___] Strongly disagree 5 | | | | | | | |  |
| Now think about the last time you had sex with ***<name>***. Would you say that it… | | | | | | | | | | | |
|  | Was very bad | 1 | | 2 | 3 | 4 | 5 | 6 | 7 | Was very good | |
|  | Was not at all what I wanted it to be | 1 | | 2 | 3 | 4 | 5 | 6 | 7 | Was very much what I wanted it to be | |
|  | Didn’t meet my expectations at all | 1 | | 2 | 3 | 4 | 5 | 6 | 7 | Exceeded my expectation | |
|  | Was not at all pleasurable | 1 | | 2 | 3 | 4 | 5 | 6 | 7 | Was very pleasurable | |
|  | Was sex that I was not into at all | 1 | | 2 | 3 | 4 | 5 | 6 | 7 | Was sex that I was totally into | |
|  | Very bad physically | 1 | | 2 | 3 | 4 | 5 | 6 | 7 | Very good physically | |
|  | Very bad emotionally | 1 | | 2 | 3 | 4 | 5 | 6 | 7 | Very good emotionally | |
|  | Was the sex act…  ***Read response options.*** | | [___] Too short 1  [___] Neither too short nor too long 2  [___] Too long 3 | | | | | | | |  |
| Now, think about your sexual experiences during the past 4 weeks… | | | | | | | | | | | |
|  | How often were you able to maintain an erection for as long as you wanted to?  ***Read response options.*** | | [___] Never or almost never 1  [___] Rarely 2  [___] Sometimes 3  [___] Usually 4  [___] Always or almost always 5 | | | | | | | |  |
|  | During sexual intercourse, how often were you able to penetrate your partner?  ***Read response options.*** | | [___] Never or almost never 1  [___] Rarely 2  [___] Sometimes 3  [___] Usually 4  [___] Always or almost always 5 | | | | | | | |  |
|  | How much have you worried about whether you could get an erection?  ***Read response options.*** | | [___] Not at all worried 1  [___] A little worried 2  [___] Somewhat worried 3  [___] Very worried 4  [___] Extremely worried 5 | | | | | | | |  |
|  | How confident were you that you could get an erection when you wanted to?  ***Read response options.*** | | [___] Not at all confident 1  [___] A little confident 2  [___] Somewhat confident 3  [___] Confident 4  [___] Very confident 5 | | | | | | | |  |
|  | How satisfied were you with the hardness of your erection?  ***Read response options.*** | | [___] Very dissatisfied 1  [___] Dissatisfied 2  [___] Equally satisfied and dissatisfied 3  [___] Satisfied 4  [___] Very satisfied 5 | | | | | | | |  |
|  | How satisfied were you with the duration of your erection?  ***Read response options.*** | | [___] Very dissatisfied 1  [___] Dissatisfied 2  [___] Equally satisfied and dissatisfied 3  [___] Satisfied 4  [___] Very satisfied 5 | | | | | | | |  |
|  | How satisfied were you with your level of sexual desire?  ***Read response options.*** | | [___] Very dissatisfied 1  [___] Dissatisfied 2  [___] Equally satisfied and dissatisfied 3  [___] Satisfied 4  [___] Very satisfied 5 | | | | | | | |  |
|  | How satisfied were you with your overall sexual activity?  ***Read response options.*** | | [___] Very dissatisfied 1  [___] Dissatisfied 2  [___] Equally satisfied and dissatisfied 3  [___] Satisfied 4  [___] Very satisfied 5 | | | | | | | |  |
|  | How much pleasure did you get from sexual activity?  ***Read response options.*** | | [___] No pleasure 1  [___] Little pleasure 2  [___] Some pleasure 3  [___] Much pleasure 4  [___] Great pleasure 5 | | | | | | | |  |
|  | How confident were you that you could satisfy your partner during sexual activity?  ***Read response options.*** | | [___] Not at all confident 1  [___] A little confident 2  [___] Somewhat confident 3  [___] Confident 4  [___] Very confident 5 | | | | | | | |  |
|  | How often did you achieve mutual satisfaction with your partner?  ***Read response options.*** | | [___] Never or almost never 1  [___] Rarely 2  [___] Sometimes 3  [___] Usually 4  [___] Always or almost always 5 | | | | | | | |  |
|  | How satisfied were you with your ability to control the timing of your ejaculation?  ***Read response options.*** | | [___] Very dissatisfied 1  [___] Dissatisfied 2  [___] Equally satisfied and dissatisfied 3  [___] Satisfied 4  [___] Very satisfied 5 | | | | | | | |  |
|  | When was the last time you had  4 or more drinks containing alcohol in one day? | | [___] Less than one week ago 1  [___] One week ago or more but less than one month ago 2  [___] One month ago or more but less than one year ago 3  [___] Never 4  [___] Decline 99 | | | | | | | |  |
|  | During the past 30 days, did you use any drugs such as marijuana, ecstasy, crack, cocaine, speed, meth, or heroin? | | [___] No 1  [___] Yes 2  [___] Decline 99 | | | | | | | |  |
|  | Has a doctor or other medical provider ever told you that you had a sexually transmitted disease? | | [___] No 1  [___] Yes 2  [___] Decline 99 | | | | | | | |  |
| Now, I’m going to read a set of statements and for each one please tell me whether you: strongly disagree, mostly disagree, somewhat disagree, neutral/no opinion, somewhat agree, mostly agree, or strongly agree. | | | | | | | | | | | |
|  | I do not hesitate to embrace new experiences if I think they can improve my health.  ***Read response options.*** | | [___] Strongly disagree 1  [___] Mostly disagree 2  [___] Somewhat disagree 3  [___] Neutral / no opinion 4  [___] Somewhat agree 5  [___] Mostly agree 6  [___] Strongly agree 7  [___] Decline 99 | | | | | | | |  |
|  | I frequently think about the health problems I may have in the future.  ***Read response options.*** | | [___] Strongly disagree 1  [___] Mostly disagree 2  [___] Somewhat disagree 3  [___] Neutral / no opinion 4  [___] Somewhat agree 5  [___] Mostly agree 6  [___] Strongly agree 7  [___] Decline 99 | | | | | | | |  |
|  | When I implement a health behavior, it's because I want to protect myself from getting sick.  ***Read response options.*** | | [___] Strongly disagree 1  [___] Mostly disagree 2  [___] Somewhat disagree 3  [___] Neutral / no opinion 4  [___] Somewhat agree 5  [___] Mostly agree 6  [___] Strongly agree 7  [___] Decline 99 | | | | | | | |  |
|  | If I succeed in reaching a health goal, this motivates me to go further.  ***Read response options.*** | | [___] Strongly disagree 1  [___] Mostly disagree 2  [___] Somewhat disagree 3  [___] Neutral / no opinion 4  [___] Somewhat agree 5  [___] Mostly agree 6  [___] Strongly agree 7  [___] Decline 99 | | | | | | | |  |
|  | I think that taking care of my health is pleasurable.  ***Read response options.*** | | [___] Strongly disagree 1  [___] Mostly disagree 2  [___] Somewhat disagree 3  [___] Neutral / no opinion 4  [___] Somewhat agree 5  [___] Mostly agree 6  [___] Strongly agree 7  [___] Decline 99 | | | | | | | |  |
|  | I see myself as someone who does my utmost to improve my health.  ***Read response options.*** | | [___] Strongly disagree 1  [___] Mostly disagree 2  [___] Somewhat disagree 3  [___] Neutral / no opinion 4  [___] Somewhat agree 5  [___] Mostly agree 6  [___] Strongly agree 7  [___] Decline 99 | | | | | | | |  |
|  | I often worry about mistakes I could make concerning my health.  ***Read response options.*** | | [___] Strongly disagree 1  [___] Mostly disagree 2  [___] Somewhat disagree 3  [___] Neutral / no opinion 4  [___] Somewhat agree 5  [___] Mostly agree 6  [___] Strongly agree 7  [___] Decline 99 | | | | | | | |  |
|  | If I see a good opportunity to improve my health, I take advantage of it right away.  ***Read response options.*** | | [___] Strongly disagree 1  [___] Mostly disagree 2  [___] Somewhat disagree 3  [___] Neutral / no opinion 4  [___] Somewhat agree 5  [___] Mostly agree 6  [___] Strongly agree 7  [___] Decline 99 | | | | | | | |  |
| Thank you very much for answering these questions. Do you have any questions for me? | | | | | | | | | | | |

**VN CSD500 Study**

**Female Follow-up Questionnaire**

| **DCF #**  **VNCSD 06** | **Staff code**  **|____||____||____||____|** | **Visit date**  **|____||____|/|____||____|/ 201|____|**  **Day Month Year** | **PIN**  **|____||____||____||____|** |
| --- | --- | --- | --- |

Now, I’m going to ask you questions for a survey. Please answer as honestly as you can. Your responses will be kept confidential and will not be shown to other persons.

| **No.** | **Question** | | **Responses** | | | | | | | | **Skip to** |
| --- | --- | --- | --- | --- | --- | --- | --- | --- | --- | --- | --- |
|  | Study visit: | | [___] 2-Month 2  [___] 4-Month 3  [___] 6-Month 4 | | | | | | | |  |
|  | Have you had any changes in your health since your last visit? | | [___] No 0  [___] Yes, specify 1  ____________________________________  [___] Decline 99 | | | | | | | |  |
|  | When was the last time you had vaginal sex with him?  By vaginal sex, I mean sex in which the penis goes into the vagina. | | [___] Today 1  [___] Yesterday 2  [___] Day before yesterday 3  [___] More than two days ago but less than one week 4  [___] One week or more but less than two weeks 5  [___] More than two weeks ago 6  [___] Decline 99 | | | | | | | |  |
|  | Did you use any type of condom for this last act with ***<name>***? | | [___] No 0  [___] Yes 1  [___] Decline 99 | | | | | | | | **🡪6** |
|  | When was the last time you had vaginal sex with him without using any type of condom for the entire act? | | [___] Today 1  [___] Yesterday 2  [___] Day before yesterday 3  [___] More than two days ago but less than one week 4  [___] One week or more but less than two weeks 5  [___] More than two weeks ago 6  [___] Never had sex with him without a condom 7  [___] Decline 99 | | | | | | | |  |
|  | Since your last study visit about two months ago, did you try to wear the study condom with your partner ***<name>***? | | [___] No, specify why not 0  ____________________________________  ____________________________________  [___] Yes 1  [___] Decline 99 | | | | | | | | **🡪10** |
|  | Since your last study visit about two months ago, have you worn another type of condom during sex with ***<name>***? | | [___] No 0  [___] Yes 1  [___] Decline 99 | | | | | | | | **🡪15** |
|  | During the past month, how often did you use any type of condom when you had sex with ***<name>***? | | [___] Always 1  [___] More than half of the time 2  [___] About half of the time 3  [___] Less than half of the time 4  [___] Never 5  [___] Decline 99 | | | | | | | |  |
|  | When was the last time that you used any type of condom when you had sex with ***<name>***? | | [___] Today 1  [___] Yesterday 2  [___] Day before yesterday 3  [___] More than two days ago but less than one week 4  [___] One week or more but less than two weeks 5  [___] More than two weeks ago 6  [___] Decline 99 | | | | | | | | **🡪12**  **🡪12**  **🡪12**  **🡪12**  **🡪12**  **🡪12**  **🡪12** |
|  | During the past month, how often did you use the study condom when you had sex with ***<name>***? | | [___] Always 1  [___] More than half of the time 2  [___] About half of the time 3  [___] Less than half of the time 4  [___] Never 5  [___] Decline 99 | | | | | | | |  |
|  | When was the last time that you used the study condom when you had sex with ***<name>***? | | [___] Today 1  [___] Yesterday 2  [___] Day before yesterday 3  [___] More than two days ago but less than one week 4  [___] One week or more but less than two weeks 5  [___] More than two weeks ago 6  [___] Decline 99 | | | | | | | |  |
| ***IF Q6 = No & Q7 = Yes:*** Think about the last time you wore a condom during sex with ***<name>***.  ***IF Q6 = Yes:*** Think about the last time you used the study condom during sex with ***<name>***. | | | | | | | | | | | |
|  | Did he use the condom for the entire act from start to finish? | | [___] Yes 1  [___] No, put on after initial penetration 2  [___] No, took off before ejaculation 3  [___] No, put on after initial penetration and took off before ejaculation 4 | | | | | | | |  |
|  | Did you have any problems with the condom?  ***Mark all that apply.*** | | [___] No 1  [___] Breakage while removing from package 2  [___] Breakage while putting on condom 3  [___] Breakage during sex or withdrawal 4  [___] Breakage while removing from penis 5  [___] Slipped down but not completely off the penis 6  [___] Slipped completely off the penis 7  [___] Other problem, specify 8  ________________________________  ________________________________ | | | | | | | |  |
|  | Did you use any other lubricant during sex with the condom? | | [___] No 0  [___] Yes, specify type 1  ________________________________ | | | | | | | |  |
| ***IF Q6 = No & Q7 = No:*** Think about the last time you had sex with ***<name>***. Would you say that it…  ***IF Q6 = No & Q7 = Yes:*** Continue to think about the last time you wore a condom during sex with ***<name>***. Would you say that it…  ***IF Q6 = Yes:*** Continue to think about the last time you used the study condom during sex with ***<name>***. Would you say that it… | | | | | | | | | | | |
|  | Was very bad | 1 | | 2 | 3 | 4 | 5 | 6 | 7 | Was very good | |
|  | Was not at all what I wanted it to be | 1 | | 2 | 3 | 4 | 5 | 6 | 7 | Was very much what I wanted it to be | |
|  | Didn’t meet my expectations at all | 1 | | 2 | 3 | 4 | 5 | 6 | 7 | Exceeded my expectation | |
|  | Was not at all pleasurable | 1 | | 2 | 3 | 4 | 5 | 6 | 7 | Was very pleasurable | |
|  | Was sex that I was not into at all | 1 | | 2 | 3 | 4 | 5 | 6 | 7 | Was sex that I was totally into | |
|  | Very bad physically | 1 | | 2 | 3 | 4 | 5 | 6 | 7 | Very good physically | |
|  | Very bad emotionally | 1 | | 2 | 3 | 4 | 5 | 6 | 7 | Very good emotionally | |
|  | Was the sex act…  ***Read response options.*** | | [___] Too short 1  [___] Neither too short nor too long 2  [___] Too long 3 | | | | | | | |  |
|  | Recall sex before you started using the study condom. Compared to then, did your partner’s erection seem…  ***Read response options.*** | | [___] More firm than usual 1  [___] The same as usual 2  [___] Less firm than usual 3 | | | | | | | |  |
|  | Did your partner’s erection seem…  ***Read response options.*** | | [___] Bigger than usual 1  [___] The same as usual 2  [___] Smaller than usual 3 | | | | | | | |  |
|  | Did your partner seem to have…  ***Read response options.*** | | [___] More pleasure than usual 1  [___] The same as usual 2  [___] Less pleasure than usual 3 | | | | | | | |  |
| ***IF Q4 = No & Q5 = No: Skip to* 🡪 Q29** | | | | | | | | | | | |
|  | Did the condom feel natural during sex?  ***Read response options.*** | | [___] Natural 1  [___] Average 2  [___] Not natural 3 | | | | | | | |  |
|  | Did the condom make penetration easier or harder than sex without a condom?  ***Read response options.*** | | [___] Easier 1  [___] No change 2  [___] Harder 3 | | | | | | | |  |
|  | Did you like using the condom?  ***Read response options.*** | | [___] Liked a lot 1  [___] Liked a little 2  [___] So-so 3  [___] Disliked a little 4  [___] Disliked a lot 5 | | | | | | | |  |
|  | When was the last time you had  4 or more drinks containing alcohol in one day? | | [___] Less than one week ago 1  [___] One week ago or more but less than one month ago 2  [___] One month ago or more but less than one year ago 3  [___] Never 4  [___] Decline 99 | | | | | | | |  |
|  | During the past 30 days, did you use any drugs such as marijuana, ecstasy, crack, cocaine, speed, meth, or heroin? | | [___] No 1  [___] Yes 2  [___] Decline 99 | | | | | | | |  |
|  | Has a doctor or other medical provider ever told you that you had a sexually transmitted disease? | | [___] No 1  [___] Yes 2  [___] Decline 99 | | | | | | | |  |
|  | Have you experienced any symptoms from wearing a study condom? | | [___] Headaches 1  [___] Faintness 2  [___] Nausea 3  [___] Loss of sensation 4  [___] Dizziness 5  [___] Skin irritation 6  [___] Other, specify 7  ____________________________________  [___] Decline 99 | | | | | | | |  |
|  | Has your partner ***<name>*** had any symptoms from wearing a study condom? | | [___] Headaches 1  [___] Faintness 2  [___] Nausea 3  [___] Loss of sensation 4  [___] Dizziness 5  [___] Skin irritation 6  [___] Other, specify 7  ____________________________________  [___] Decline 99 | | | | | | | |  |
| Thank you very much for answering these questions. Do you have any questions for me? | | | | | | | | | | | |

**VN CSD500 Study**

**Male Follow-up Questionnaire**

| **DCF #**  **VNCSD 07** | **Staff code**  **|____||____||____||____|** | **Visit date**  **|____||____|/|____||____|/ 201|____|**  **Day Month Year** | **PIN**  **|____||____||____||____|** |
| --- | --- | --- | --- |

Now, I’m going to ask you questions for a survey. Please answer as honestly as you can. Your responses will be kept confidential and will not be shown to other persons.

| **No.** | **Question** | | **Responses** | | | | | | | | **Skip to** |
| --- | --- | --- | --- | --- | --- | --- | --- | --- | --- | --- | --- |
|  | Study visit: | | [___] 2-Month 2  [___] 4-Month 3  [___] 6-Month 4 | | | | | | | |  |
|  | Have you had any changes in your health since your last visit? | | [___] No 0  [___] Yes, specify 1  ____________________________________  [___] Decline 99 | | | | | | | |  |
|  | When was the last time you had vaginal sex with ***<name>***?  By vaginal sex, I mean sex in which the penis goes into the vagina. | | [___] Today 1  [___] Yesterday 2  [___] Day before yesterday 3  [___] More than two days ago but less than one week 4  [___] One week or more but less than two weeks 5  [___] More than two weeks ago 6  [___] Decline 99 | | | | | | | |  |
|  | Did you use any type of condom for this last act with ***<name>***? | | [___] No 0  [___] Yes 1  [___] Decline 99 | | | | | | | | **🡪6** |
|  | When was the last time you had vaginal sex with her without using a condom for the entire act? | | [___] Today 1  [___] Yesterday 2  [___] Day before yesterday 3  [___] More than two days ago but less than one week 4  [___] One week or more but less than two weeks 5  [___] More than two weeks ago 6  [___] Never had sex with partner without using a condom 7  [___] Decline 99 | | | | | | | |  |
|  | Did you try to wear the study condom with your partner ***<name>***? | | [___] No, specify why not 0  ____________________________________  ____________________________________  [___] Yes 1  [___] Decline 99 | | | | | | | | **🡪8** |
|  | Since you joined this study about 6 months ago, have you worn another type of condom during sex with ***<name>***? | | [___] No 0  [___] Yes 1  [___] Decline 99 | | | | | | | | **🡪13**  **🡪10**  **🡪10** |
|  | During the past month, how often did you use the study condom when you had sex with ***<name>***? | | [___] Always 1  [___] More than half of the time 2  [___] About half of the time 3  [___] Less than half of the time 4  [___] Never 5  [___] Decline 99 | | | | | | | |  |
|  | When was the last time that you used the study condom when you had sex with ***<name>***? | | [___] Today 1  [___] Yesterday 2  [___] Day before yesterday 3  [___] More than two days ago but less than one week 4  [___] One week or more but less than two weeks 5  [___] More than two weeks ago 6  [___] Decline 99 | | | | | | | |  |
| ***IF Q6 = No & Q7 = Yes:*** Think about the last time you wore a condom during sex with ***<name>***.  ***IF Q6 = Yes:*** Think about the last time you used the study condom during sex with ***<name>***. | | | | | | | | | | | |
|  | Did you use the condom for the entire act from start to finish? | | [___] Yes 1  [___] No, put on after initial penetration 2  [___] No, took off before ejaculation 3  [___] No, put on after initial penetration and took off before ejaculation 4 | | | | | | | |  |
|  | Did you have any problems with the condom?  ***Mark all that apply.*** | | [___] No 1  [___] Breakage while removing from package 2  [___] Breakage while putting on condom 3  [___] Breakage during sex or withdrawal 4  [___] Breakage while removing from penis 5  [___] Slipped down but not completely off the penis 6  [___] Slipped completely off the penis 7  [___] Other problem, specify 8  ________________________________  ________________________________ | | | | | | | |  |
|  | Did you use any other lubricant during sex with the condom? | | [___] No 0  [___] Yes, specify type 1  ________________________________ | | | | | | | |  |
| ***IF Q6 = No & Q7 = No:*** Think about the last time you had sex with ***<name>***. Would you say that it…  ***IF Q6 = No & Q7 = Yes:*** Continue to think about the last time you wore a condom during sex with ***<name>***. Would you say that it…  ***IF Q6 = Yes:*** Continue to think about the last time you used the study condom during sex with ***<name>***. Would you say that it… | | | | | | | | | | | |
|  | Was very bad | 1 | | 2 | 3 | 4 | 5 | 6 | 7 | Was very good | |
|  | Was not at all what I wanted it to be | 1 | | 2 | 3 | 4 | 5 | 6 | 7 | Was very much what I wanted it to be | |
|  | Didn’t meet my expectations at all | 1 | | 2 | 3 | 4 | 5 | 6 | 7 | Exceeded my expectation | |
|  | Was not at all pleasurable | 1 | | 2 | 3 | 4 | 5 | 6 | 7 | Was very pleasurable | |
|  | Was sex that I was not into at all | 1 | | 2 | 3 | 4 | 5 | 6 | 7 | Was sex that I was totally into | |
|  | Very bad physically | 1 | | 2 | 3 | 4 | 5 | 6 | 7 | Very good physically | |
|  | Very bad emotionally | 1 | | 2 | 3 | 4 | 5 | 6 | 7 | Very good emotionally | |
|  | Was the sex act…  ***Read response options.*** | | [___] Too short 1  [___] Neither too short nor too long 2  [___] Too long 3 | | | | | | | |  |

| Now, think about your sexual experiences during the past 4 weeks… | | | |
| --- | --- | --- | --- |
|  | How often were you able to maintain an erection for as long as you wanted to?  ***Read response options.*** | [___] Never or almost never 1  [___] Rarely 2  [___] Sometimes 3  [___] Usually 4  [___] Always or almost always 5 |  |
|  | During sexual intercourse, how often were you able to penetrate your partner?  ***Read response options.*** | [___] Never or almost never 1  [___] Rarely 2  [___] Sometimes 3  [___] Usually 4  [___] Always or almost always 5 |  |
|  | How much have you worried about whether you could get an erection?  ***Read response options.*** | [___] Not at all worried 1  [___] A little worried 2  [___] Somewhat worried 3  [___] Very worried 4  [___] Extremely worried 5 |  |
|  | How confident were you that you could get an erection when you wanted to?  ***Read response options.*** | [___] Not at all confident 1  [___] A little confident 2  [___] Somewhat confident 3  [___] Confident 4  [___] Very confident 5 |  |
|  | How satisfied were you with the hardness of your erection?  ***Read response options.*** | [___] Very dissatisfied 1  [___] Dissatisfied 2  [___] Equally satisfied and dissatisfied 3  [___] Satisfied 4  [___] Very satisfied 5 |  |
|  | How satisfied were you with the duration of your erection?  ***Read response options.*** | [___] Very dissatisfied 1  [___] Dissatisfied 2  [___] Equally satisfied and dissatisfied 3  [___] Satisfied 4  [___] Very satisfied 5 |  |
|  | How satisfied were you with your level of sexual desire?  ***Read response options.*** | [___] Very dissatisfied 1  [___] Dissatisfied 2  [___] Equally satisfied and dissatisfied 3  [___] Satisfied 4  [___] Very satisfied 5 |  |
|  | How satisfied were you with your overall sexual activity?  ***Read response options.*** | [___] Very dissatisfied 1  [___] Dissatisfied 2  [___] Equally satisfied and dissatisfied 3  [___] Satisfied 4  [___] Very satisfied 5 |  |
|  | How much pleasure did you get from sexual activity?  ***Read response options.*** | [___] No pleasure 1  [___] Little pleasure 2  [___] Some pleasure 3  [___] Much pleasure 4  [___] Great pleasure 5 |  |
|  | How confident were you that you could satisfy your partner during sexual activity?  ***Read response options.*** | [___] Not at all confident 1  [___] A little confident 2  [___] Somewhat confident 3  [___] Confident 4  [___] Very confident 5 |  |
|  | How often did you achieve mutual satisfaction with your partner?  ***Read response options.*** | [___] Never or almost never 1  [___] Rarely 2  [___] Sometimes 3  [___] Usually 4  [___] Always or almost always 5 |  |
|  | How satisfied were you with your ability to control the timing of your ejaculation?  ***Read response options.*** | [___] Very dissatisfied 1  [___] Dissatisfied 2  [___] Equally satisfied and dissatisfied 3  [___] Satisfied 4  [___] Very satisfied 5 |  |
| ***IF Q6 = No & Q7 = No: Skip to* 🡪 Q45** | | | |
|  | Was the condom easy or hard to put on?  ***Read response options.*** | [___] Very easy 1  [___] Easy 2  [___] Neither easy nor difficult 3  [___] Difficult 4  [___] Very difficult 5 |  |
|  | Was the condom easy or hard to keep on during sex?  ***Read response options.*** | [___] Very easy 1  [___] Easy 2  [___] Neither easy nor difficult 3  [___] Difficult 4  [___] Very difficult 5 |  |
|  | Was the condom easy or hard to remove?  ***Read response options.*** | [___] Very easy 1  [___] Easy 2  [___] Neither easy nor difficult 3  [___] Difficult 4  [___] Very difficult 5 |  |
|  | How did the condom fit?  ***Read response options.*** | [___] Very good 1  [___] Good 2  [___] Average 3  [___] Below average 4  [___] Poor 5 |  |
|  | Did the condom feel natural during sex?  ***Read response options.*** | [___] Natural 1  [___] Average 2  [___] Not natural 3 |  |
|  | Did the condom make penetration easier or harder than sex without a condom? | [___] Easier 1  [___] No change 2  [___] Harder 3 |  |
|  | Did you or your partner experience pain during sex because of the condom? | [___] No 0  [___] Yes 1 |  |
|  | Did you or your partner experience burning, itching or irritation when using the condom? | [___] No 0  [___] Yes, I experienced this 1  [___] Yes, my partner experienced this 2  [___] Yes, we both experienced this 3 |  |
|  | Did you or your partner use lubrication when using the condom? | [___] No 0  [___] Yes 1 |  |
|  | Was sex with the condom shorter or last longer than sex without a condom? | [___] Shorter 1  [___] No change 2  [___] Longer 3 |  |
|  | Did you like using the condom?  ***Read response options.*** | [___] Like a lot 1  [___] Like a little 2  [___] Didn’t matter 3  [___] Dislike a little 4  [___] Dislike a lot 5 |  |
|  | How did sex with the condom feel to you compared with sex without a condom?  ***Read response options.*** | [___] A lot better 1  [___] A little better 2  [___] No different 3  [___] A little worse 4  [___] A lot worse 5  [___] Never had sex without a condom 6 |  |
|  | When was the last time you had  4 or more drinks containing alcohol in one day? | [___] Less than one week ago 1  [___] One week ago or more but less than one month ago 2  [___] One month ago or more but less than one year ago 3  [___] Never 4  [___] Decline 99 |  |
|  | During the past 30 days, did you use any drugs such as marijuana, ecstasy, crack, cocaine, speed, meth, or heroin? | [___] No 1  [___] Yes 2  [___] Decline 99 |  |
|  | Has a doctor or other medical provider ever told you that you had a sexually transmitted disease? | [___] No 1  [___] Yes 2  [___] Decline 99 |  |
| Thank you very much for answering these questions. Do you have any questions for me? | | | |

**VN CSD500 Study**

**Study Discontinuation Form**

| **DCF #**  **VNCSD 08** | | | **Staff code**  **|____||____||____||____|** | **Visit date**  **|____||____|/|____||____|/ 201|____|**  **Day Month Year** | | **PIN**  **|____||____||____||____|** | |
| --- | --- | --- | --- | --- | --- | --- | --- |
|  |  | | | | | | |
|  | **No.** | **Questions** | | | **Coding categories** | | **Skip to** |
|  | **1.** | Reason for discontinuation: | | | [___] Completed study 1  [___] Lost to follow-up 2  [___] Discontinued, personal reason not related to the study: 3  [___] Discontinued, personal reason related to the study, specify: 4  __________________________  [___] Discontinued, medical reason not related to the study 5  [___] Discontinued, medical reason related to the study, specify: 6  __________________________  [___] Discontinued, not eligible for study due to pregnancy at enrollment 7  [___] Other, specify: 8  __________________________ | |  |

**VN CSD500 Study**

**Interim Visit Form**

| **DCF #**  **VNCSD 09** | **Staff code**  **|____||____||____||____|** | **Visit date**  **|____||____|/|____||____|/ 201|____|**  **Day Month Year** | **PIN**  **|____||____||____||____|** |
| --- | --- | --- | --- |

| **No.** | **Question** | **Coding categories** | **Skip to** |
| --- | --- | --- | --- |
| **1.** | Reason for participant’s visit:  ***Mark all that apply.*** | [___] Problem with condom 1  [___] Other medical problem 2  [___] Questions about study 3  [___] Study discontinuation 4  [___] Other, specify 5  __________________________ |  |

**VN CSD500 Study**

**Adverse Event Form**

| **DCF #**  **VNCSD 10** | **Staff code**  **|____||____||____||____|** | **Visit date**  **|____||____|/|____||____|/ 201|____|**  **Day Month Year** | **PIN**  **|____||____||____||____|** |
| --- | --- | --- | --- |

| **No.** | **Question** | **Coding categories** | **Skip to** |
| --- | --- | --- | --- |
|  | Does the participant have any known medical conditions that may have contributed to the adverse event? | [___] No, 0  [___] Yes, specify 1  __________________________  __________________________  __________________________  __________________________ |  |
|  | Describe the adverse event.  A diagnosis is preferred. If no diagnosis, describe signs or symptoms. | __________________________  __________________________  __________________________  __________________________  __________________________ |  |
|  | How was the adverse event identified?  ***Mark all that apply.*** | [___] Participant report 1  [___] Clinician 2  [___] Other, specify 3  __________________________  [___] Don’t know 4 |  |
|  | When did the adverse event begin? | **|____||____| / |____||____| /201|____|**  Day Month Year |  |
|  | Has the adverse event resolved? | [___] No 0  [___] Yes 1  [___] Don’t know 88 | **🡪7**  **🡪7** |
|  | When did the adverse event resolve? | **|____||____| / |____||____| /201|____|**  Day Month Year |  |
|  | What is the current status? | [___] Resolved without sequelae 1  [___] Resolved with sequalae, specify: 2  __________________________ |  |
|  | Was the adverse event expected? | [___] No 0  [___] Yes 1  [___] Don’t know 88 |  |
|  | Is this a serious adverse event? | [___] No 0  [___] Yes 1 | **🡪11** |
|  | What is the category of serious adverse event?  ***Mark all that apply.*** | [___] Death  [___] Potentially life-threatening  [___] Immediately life-threatening  [___] Persistent or significant disability or incapacity  [___] Resulted in congenital anomaly  [___] Serious as assessed by the investigator  [___] Required brief hospitalization (<48 hours)  [___] Required prolonged hospitalization (>=48 hours)  [___] Other, specify:  __________________________  __________________________ |  |
|  | Was the adverse event related to study participation? | [___] Definitely related 1  [___] Probably related 2  [___] Possibly related 3  [___] Probably not or unlikely to be related 4  [___] Not related 5 | **🡪13**  **🡪13** |
|  | How was the adverse event related to study participation? | __________________________  __________________________  __________________________  __________________________ |  |
|  | How was the event managed?  ***Mark all that apply.*** | [___] None 1  [___] Observation 2  [___] Prescription or medicine provided, specify: 3  __________________________  __________________________  [___] Required brief hospitalization (<48 hours) 4  [___] Required prolonged hospitalization (>=48 hours) 5  [___] Other, specify 6  __________________________  __________________________ |  |
